# Evolution of research trends in artificial intelligence for breast cancer diagnosis and prognosis over the past two decades: A bibliometric analysis

**DOI:** 10.3389/fonc.2022.854927

**Published:** 2022-09-23

**Authors:** Asif Hassan Syed, Tabrej Khan

**Affiliations:** ^1^ Department of Computer Science, Faculty of Computing and Information Technology Rabigh (FCITR), King Abdulaziz University, Jeddah, Saudi Arabia; ^2^ Department of Information Systems, Faculty of Computing and Information Technology Rabigh (FCITR), King Abdulaziz University, Jeddah, Saudi Arabia

**Keywords:** artificial intelligence, breast cancer, diagnosis and prognosis, Bibliometrix analysis, knowledge structures

## Abstract

**Objective:**

In recent years, among the available tools, the concurrent application of Artificial Intelligence (AI) has improved the diagnostic performance of breast cancer screening. In this context, the present study intends to provide a comprehensive overview of the evolution of AI for breast cancer diagnosis and prognosis research using bibliometric analysis.

**Methodology:**

Therefore, in the present study, relevant peer-reviewed research articles published from 2000 to 2021 were downloaded from the Scopus and Web of Science (WOS) databases and later quantitatively analyzed and visualized using Bibliometrix (R package). Finally, open challenges areas were identified for future research work.

**Results:**

The present study revealed that the number of literature studies published in AI for breast cancer detection and survival prediction has increased from 12 to 546 between the years 2000 to 2021. The United States of America (USA), the Republic of China, and India are the most productive publication-wise in this field. Furthermore, the USA leads in terms of the total citations; however, hungry and Holland take the lead positions in average citations per year. Wang J is the most productive author, and Zhan J is the most relevant author in this field. Stanford University in the USA is the most relevant affiliation by the number of published articles. The top 10 most relevant sources are Q1 journals with PLOS ONE and computer in Biology and Medicine are the leading journals in this field. The most trending topics related to our study, transfer learning and deep learning, were identified.

**Conclusion:**

The present findings provide insight and research directions for policymakers and academic researchers for future collaboration and research in AI for breast cancer patients.

## Introduction

Breast cancer is the most commonly diagnosed cancer among women in most countries (159 of 185 countries), with an estimated 2.3 million women diagnosed with breast cancer in 2020. Moreover, breast cancer is the leading cause of cancer death in women in 110 countries, with 685000 deaths globally (1). However, early detection and prognosis prediction, which involves explicitly estimating the relapse of breast tumors and predicting the 5-year survival rate of the breast cancer patient, can significantly improve patient outcomes (2, 3). In this context, several developed countries have employed extensive mammography, Magnetic Resonance Imaging, breast ultrasound, and thermography-based screening programs for earlier breast cancer (4, 5). However, one of the significant challenges lies in interpreting these images generated by such techniques. In addition, the precision and accuracy achieved by even the best clinicians in detecting breast cancer using mammography vary widely, thus leaving room for further improvements (6, 7). In this context, in the 1990s, Computer-aided software detection was introduced for mammography, and several software assistive applications have been approved for medical use. However, despite initial promising implementations, the software tools of the 1990’s era could not significantly improve the performance of mammography readers in real-world scenarios (7–11).

Over the past few years, AI’s potential in precision oncology has uniquely poised to handle the errors associated with medical image analysis (12–19). AI is centered on developing high-level algorithms to execute complex tasks in clinical settings in radiology to quickly and effectively aid in interpreting image data. The main objective of applying AI to image analysis is to reveal a visual pattern from image data and assist clinicians and mammogram experts in formulating effective clinical decisions about breast cancer detection and survival prediction. In recent years, the field of AI in breast cancer research has seen a resurgence owed to the commendable performances of Deep Learning (DL) in detecting breast cancer and further predicting the 5-years survival of breast cancer using mammography. Studies have shown the capacity of DL to be at par, or in some cases, exceed the performance of human experts in medical–image analysis for the diagnosis and prognosis of breast cancer (20, 21). As the scarcity of mammography experts threatens the availability and sufficiency of breast-screening services worldwide, AI agents’ unique precision and accuracy in an image- analysis could enhance the access to high-quality diagnosis and prognosis of breast cancer. Therefore, the prospects of AI in facilitating clinicians in clinical decision-making and managing breast cancer are manifold and ever-expanding. As the applications of AI in breast cancer diagnosis and prognosis grow, it becomes necessary to comprehend the ongoing research setting and future research trajectory. However, the AI-based research in breast cancer detection and survival prediction does not explore inherent development rules and current research trends and discuss the challenges that the AI will face in diagnosing and prognosis of Breast Cancer. Therefore, to achieve the goal, the present study aims to review the existing research articles through bibliometric analysis to learn about the global progress and trends in the application of AI for breast cancer detection and survival prediction. Bibliometric analysis is a quantitative analysis of research publications to describe the trends in academic literature, the contributions of journals and authors, nations’ productivity in a particular research area, and info regarding research collaborations and cooperation (22–24). In addition, the bibliometric analysis enables monitoring of the patterns and trends of effectual publications in several areas, including healthcare research (25).

**Figure 1:**
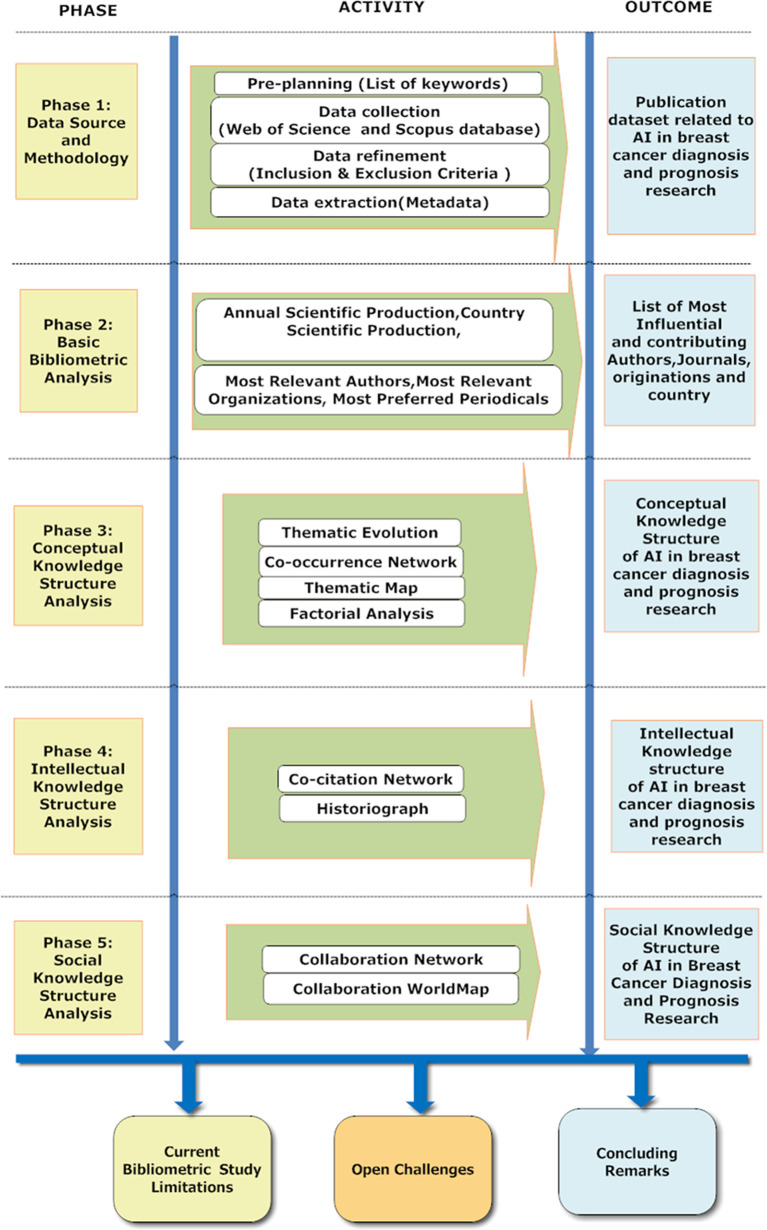
Bibliometric Process for Reviewing the AI publications in breast cancer diagnosis and prognosis research from 2000 to 2021.

Thus, the current bibliometric analysis findings will help researchers, governments, and entrepreneurs understand the Development of AI research in breast cancer diagnosis and prognosis in the last two decades. For research scholars and scientists, the present study results will be helpful to know about the important journals and understand the thematic trends of AI in breast cancer diagnosis and prognosis research. Our study will help governments devise more proficient present and future action strategies centered on AI research and development evolution trends in breast cancer diagnosis and prognosis. In the context of entrepreneurs, the results will help scree the most contributing research organizations toward AI for breast cancer research and also develop a competitive AI market for developing AI applications for breast cancer detection and survival prediction after understanding the collaboration networks of the AI in breast cancer diagnostic and prognostic research area. Moreover, the current study is the first to quantitatively analyze the hot research domains of breast cancer research and the application of AI in cancer detection and survival prediction. Our study portrays the impact of scientificc information by indicating gaps and presenting a meaningful path for future research in AI for breast cancer detection and survival prediction. An overview of the systematic review of AI’s application in breast cancer detection and survival prediction includes eight distinct phases, as shown in **Figure 1**. As shown in **Figure 1**, Phase-1 presents the data source and methodology; Phase-2 offers the fundamental bibliometric analysis; Phase-3 shows the conceptual knowledge structure analysis; Phase-4 describes the intellectual knowledge structure analysis; Phase-5 describes the social knowledge structure analysis; Phase-6 lists the current bibliometric limitations; Phase-7 describes the open challenges of AI in breast cancer diagnosis and prognosis research; and finally, Phase-8 describes the concluding remarks.

## Materials and methods

### Methodology and data sources

#### Pre-planning

In the pre-planning stage, search queries were selected as tabulated in **Supplementary Table S1**. The search queries were categorized as 1) key search terms and 2) a combination of key search terms with breast cancer and search items related to the prediction and classification of breast cancer. The key search terms included AI, Machine Learning (ML), and names of different supervised and unsupervised algorithms as tabulated in **Supplementary Table S1**. The second search terms, as tabulated in **Supplementary Table S1**, included a combination of search queries in association with “breast cancer and detection,” “breast cancer and classification,” “breast cancer and prognosis detection,” “breast cancer and mortality risk,” “breast cancer and survival,” “breast cancer and prediction,” and finally “breast cancer and microarray gene expression.” A subset of crucial search queries and different combinations of key search terms were selected based on the relevance of the search criteria to AI and its application in breast cancer diagnosis and prognosis research. Our search scope expanded but remained focused on breast cancer by searching literature using key search terms combined with breast cancer and search items that include the word prediction, classification, diagnosis, and prognosis of breast cancer. The idea of adding breast cancer and microarray gene expression criterion with the key search items, namely AI and ML, is to explore and analyze the application of AI and ML in breast cancer research using microarray gene expression data. Since microarray gene expression data plays a significant role in understanding the role of different gene biomarkers in the pathophysiology of breast cancer disease initiation and progression. Thereby employing AI and ML techniques, the most relevant/informative breast cancer gene biomarkers can be screened, and subsequently, classification and deep learning models can be constructed to predict and classify the disease’s different stages. Therefore, the involving gene microarray data with AI helps us understand the evolving role of AI in breast cancer severity, mortality, and survival predictions across the past two decades.

In addition, appropriate research questions were formulated as tabulated in **Supplementary Table S2** to provide a comprehensive overview of the knowledge structure and bibliometric and statistical techniques to evaluate the role of AI research in breast cancer detection and survival prediction from the year 2000 to 2021.

#### Data collection

In the data collection stage, we systematically searched academic articles in WOS core collection and Scopus databases from 1^st^ January 2000 to 31^st^ September 2021 that involved AI’s application in breast cancer detection and survival prediction research. The keywords used for the data retrieval are tabulated in **Supplementary Table S1**. In addition, research articles and review papers written in English were included in the present study. From Scopus 10161 academic publications and ISI WOS, 7277 research publications were retrieved for analysis.

#### Data refinement

Further, in the data refinement stage, the publications retrieved from WOS and Scopus were refined based on the exclusion criteria tabulated in **Supplementary Table S2**. In addition, we excluded studies published as books, editorials, letters, conference papers, and academic publications not published in the English language were excluded from our systematic bibliometric review. Lastly, the refined list of publications obtained from Scopus (1737) and WOS (1841) was combined by removing the redundant publications. Therefore, after the refinement process, the total number of articles was reduced to 2641. A systematic workflow of the selection criteria for data collection and refinement is shown in **Supplementary Figure S1** and **Supplementary Table S3**.

#### Data extraction

We retrieved the metadata from Scopus and WOS as a bibliographic information file (.bib file). The data exported included: (a) authors/editors, (b) authors full name, (c) title, (d) source, (e) authors’ keywords, (f) keywords plus, (g) abstracts, (h) authors affiliations, (i) corresponding authors affiliation, (j) cited references, (j) total citations, (k) highly cited (l) usage counts (m) publication year, (n) DOI, (o) subject category, (p) author identifiers, (q) languages, and (r) funding agencies.

#### Bibliometric data analysis

The bibliometric analysis enables a researcher to record, access objectively, and process hundreds or thousands of publications to profoundly summarize recent trends in scientific publications in a discipline or specifically in a research area. In the present study, a bibliometric analysis of publications related to the evolution of AI research in breast cancer diagnosis and prognosis from 2000 to date is performed to address the six major queries as tabulated in **Supplementary Table S2**. The bibliometric data analysis was conducted using biblioshiny (26) to represent the publication patterns and the research trends in implementing AI on breast cancer diagnosis and prognosis. In addition, we intend to statistically explore and evaluate the scientific knowledge structure through the current bibliometric analysis. The basic knowledge structure of a research field can be categorized into three parts such as:

1. Conceptual structure (what literature talks about central themes and trends related to a specific research field)

2. Intellectual structure (How the work of an author influences a given scientific community)

3. Social structure (how authors, institutions, and countries interact with each other)

Firstly, the conceptual structure is explored statistically using thematic mapping (27), thematic evolution, co-occurrence network, and factorial analysis. Secondly, the intellectual knowledge structure was assessed by performing co-citation network analysis (28) and historiography (29). Finally, the social knowledge structure was reviewed based on the collaboration network and collaboration world map. Therefore, upon analyzing the conceptual, intellectual, and social structure, we can understand the knowledge structure of the application of AI in breast cancer diagnosis and prognosis during the last two decades. Thus upon analyzing the knowledge structure of AI in breast cancer in the previous two decades, we will understand the current accomplishments and future open challenges in implementing AI for breast cancer diagnosis and prognosis.

## Results

### Annual scientific production

**Figure 2:**
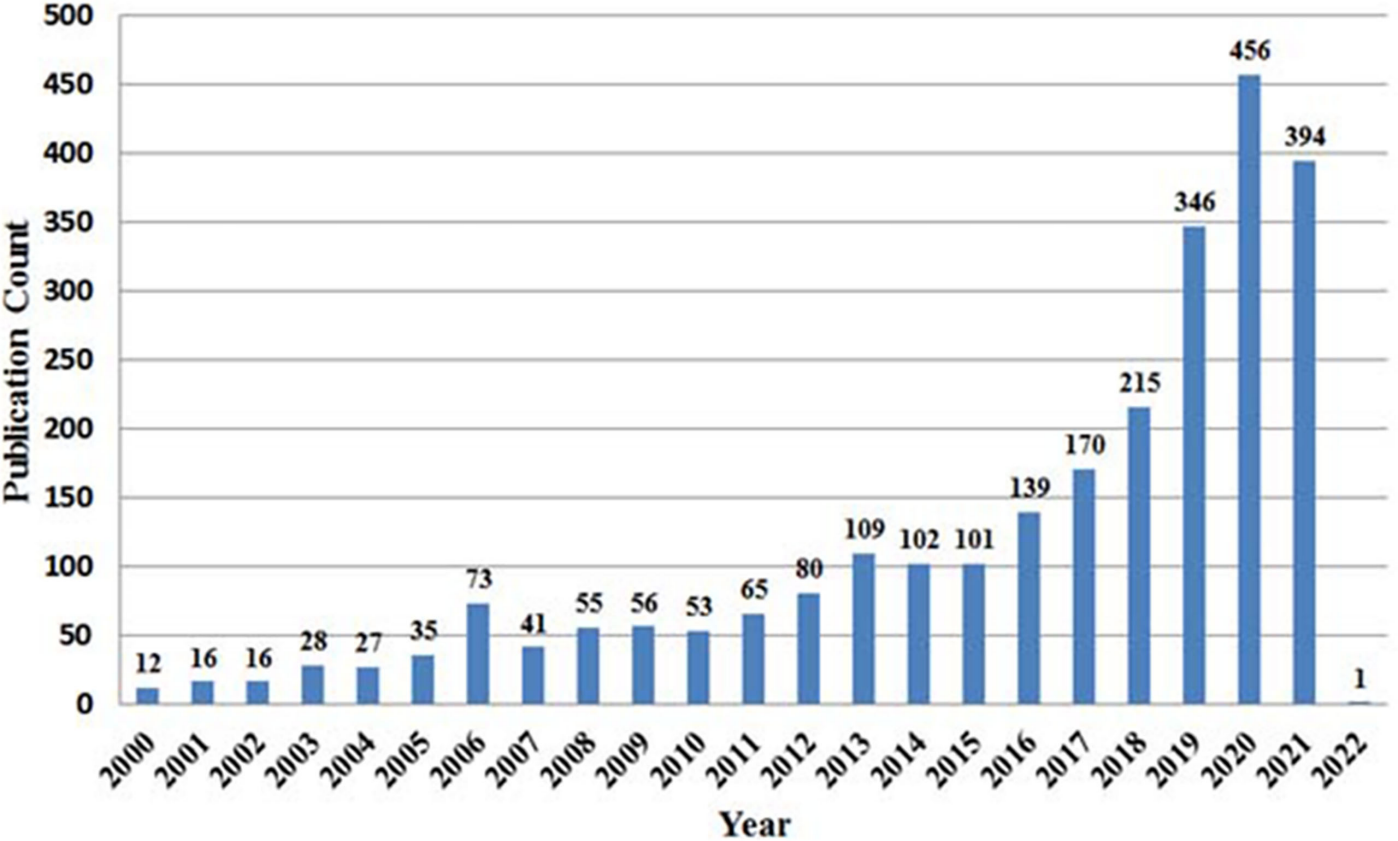
Yearly Publication of AI application in breast cancer diagnosis and prognosis research.

The number of publications from 2000 to 2021 shows the evolution of the research and trends in AI for breast cancer diagnosis and prognosis. The current study uses WOS and Scopus databases to mine 2641 academic publications from 2000 to 2021 using the query listed in **Supplementary Table S1**. As shown in **Figure 2**, the yearly scientific publication presents variations in scientific contribution in the research field mentioned above within a specified time duration. The analysis shows that the global scientific publication trends in AI for breast cancer diagnosis and prognosis peaked in 2019-2021, with 2020 being the most productive year (456 scientific publications). Thus, the increasing frequency of international academic literature in the last six years (2016 to 2021) depicts a growing intensity of research in AI for breast cancer diagnosis and prognosis. Therefore, we can presume that the research in AI for breast cancer diagnosis and prognosis has attracted the most attention of researchers during the last decade (2011-2021).

#### Most relevant authors

The current paragraph highlights the most prolific researchers in the field of AI for breast cancer detection and survival predictions in terms of the number of publications in this area and the impact of their publications. **Table 1** shows the 15 most prolific authors with their number of publications, total citations, and corresponding h-index. As is evident from **Table 1**, Zang, Y from Henan Polytechnic University, Jiaozuo, China, has the most number of publications, i.e., 31, closely followed by Wang Y from Hangzhou Dianzi University, Hangzhou, China, Li Y from Chongquing University/Third Military Medical University, Chongqing and Zhang J from Zhejiang Cancer Hospital, Zhejiang Hangzhou, China with 28 publication each author. However, regarding the impact of these publications in terms of total citations, Chen H has the highest citations with 1302 citations, followed by Madabhushi, A, Rangayan, R with 1233 and 1225 citations, respectively. Furthermore, Chen H and Zhang Y is the most contributing author with an h-index of 13, followed by Rangayyan R with 12, Zhang X, and Wang Y with 12 each. Thus, the table suggests that Zang Y, with the highest number of publications, is the most contributing researcher in AI for breast cancer detection and prognosis predictions.

**Table 1:** Tabulation of the 15 most prolific authors with their number of publications (NP), Total Citation (TC), and corresponding h-index (Note the authors are ranked based on h-index and h-index obtained from biblioshiny).

Rank	Element	H_index	TC	NP
1.	CHEN H	13	1302	17
2.	ZHANG Y	13	445	31
3.	RANGAYYAN R	12	1225	13
4.	ZHANG X	12	791	21
5.	WANG Y	12	666	28
6.	ZHANG J	12	444	28
7.	WANG J	11	663	24
8.	YANG Y	10	1107	15
9.	CHEN X	10	1080	13
10.	LIU J	10	648	18
11.	LI Y	10	565	28
12.	CHEN Y	10	418	20
13.	SILVA A	10	377	18
14.	MADABHUSHI A	9	1233	10
15.	POLAT K	9	654	10

#### Most relevant organizations

The top 10 most contributing/relevant organizations in AI for breast cancer detection and survival prediction research are represented in **Supplementary Figure S2**. As per **Supplementary Figure S2**, there are five most productive organizations, among which Stanford University, USA, is the topmost productive organization with 38 publications, followed by National Taiwan University, Taiwan, with 37 publications, Sun Yat-sen University, China, with 32 publications, University of Malaya, Malaysia with 32 publication and Sichuan University, China, with 30 publications. Moreover, it is remarkable that out of the top 10 organizations globally, four organizations are from China.

#### Country scientific production

The top 20 contributing countries in AI for breast cancer detection and survival prediction are shown in **Table 2**. The data tabulated in **Table 2** includes the total article published in the given field, total citations, and the average article citations. It appears from **Table 2** that there are only two countries (China and USA) producing more than one thousand publications in the AI for breast cancer detection and survival prediction from the year 2000 to 2021. As per **Table 2**, the Republic of China is the top scientific productive country with 1217 publications, followed by the USA with 1100 publications, and India with 690 publications in AI for breast cancer detection and survival prediction research. The USA is the most influential country with 13015 citations, followed by China and United Kingdom (UK) with 9375 and 3166 citations. Surprisingly, the Netherlands is in twenty positions in terms of publication numbers. However, the average article citation in the Netherland is 82.26, which is the highest among the top twenty countries. Thereby, we can conclude that Netherland significantly impacts research in AI in breast cancer diagnosis and prognosis.

**Table 2:** Tabulation of the top 20 contributing countries in AI for breast cancer detection and survival prediction (Note that the countries are ranked based on the number of publications).

Region	Number of Publications	Total Citations	Average Article Citations
CHINA	1217	9375	19.7
USA	1100	13015	34.43
INDIA	690	3153	8.64
UK	273	3166	39.09
CANADA	217	1318	20.28
SPAIN	201	2581	51.62
GERMANY	191	2562	45.75
SOUTH KOREA	189	1445	19.01
IRAN	158	1438	19.43
TURKEY	145	2506	30.19
ITALY	139	822	16.12
AUSTRALIA	125	1819	34.32
MALAYSIA	121	617	10.82
EGYPT	115	1302	21
PAKISTAN	112	532	12.98
SAUDI ARABIA	106	385	9.17
FRANCE	98	493	22.41
BRAZIL	97	908	19.32
SINGAPORE	73	877	38.13
NETHERLANDS	71	2221	82.26

#### Most preferred periodicals

The number of publications in terms of Bradford law called the core sources the nucleus of journals, mainly devoted to the given research area. It appears from **Supplementary Figure S3** that the top ten journals, as tabulated in **Table 3**, form the core of journals publishing about a third of the documents of the entire collection. The leading ten relevant periodicals that published one or more articles included in our bibliographic collection are tabulated in **Supplementary Table S4**. It is noteworthy that PLOS ONE, with 96 articles, is the most preferred publishing venue, followed by Computers in Biology and Medicine and Expert Systems With Application with 86 and 81 articles. In terms of the *H*-index, which is a journals number of published articles (*h*), each of which has been cited by other papers at least h time, Expert System with Applications with an h-index of 36 and with amazingly 4230 total citations is the most leading journal, followed by IEEE Transactions On Medical Imaging (*h*-index = 32, TC = 4223). Artificial Intelligence in Medicine (*H*-index = 28, TC = 2837), Computers in Biology and Medicine (*H*-index = 28, TC = 2147) and BMC Bioinformatics (*H* -index = 24, TC = 3114) being other most prominent journals publishing in the area of AI in breast cancer detection and survival predictions.

**Table 3:** Top 10 preferred periodicals for AI in breast cancer detection and survival prediction research from the year 2000 to 2021 (The journals are ranked based on the *H*-index).

Sources	Articles	H-index	Total Citations
PLOS ONE	96	26	2242
Computers In Biology And Medicine	86	28	2147
Expert Systems With Applications	81	36	4230
IEEE Access	80	13	627
Scientific Reports	77	17	1736
BMC Bioinformatics	72	24	3114
Computer Methods And Programs In Biomedicine	66	23	1615
Artificial Intelligence In Medicine	64	28	2837
Neurocomputing	62	25	2529
IEEE Transactions On Medical Imaging	56	32	4223

### Highly cited research publications in AI for breast cancer detection and survival predictions

The topmost ten highly local cited (Local citation measures the impact of documents in the analyzed collection) research publications within AI for the given research area published between 2000 to 2021 are tabulated in **Table 4**. For example, Delen D 2005 (30) published an article titled “*Predicting breast cancer survivability: a comparison of three data mining methods*” published in “AI in Medicine” is the most locally cited article with 65 local citations and 539 global citations, respectively. Akay MF 2009 (31), with the article entitled “*Support vector machines (SVM) combined with feature selection for breast cancer diagnosis*” published in Expert System and applications, was the second most influential paper with 64 local citations and 367 global citations. Also, Zheng B 2014 (32) published an article entitled “*Breast cancer diagnosis based on feature extraction* using a hybrid of K-means and SVM algorithms” that got 58 local citations and 214 global citations. Finally, Kooi T 2017 (33) published an article entitled “*Large scale DL for computer-aided detection of mammographic lesions* **”** with 55 local and 387 global citations. Therefore, as shown in **Table 4**, these authors are the most influential authors contributing to AI for breast cancer detection and survival prediction research from 2000 to 2021.

**Table 4:** List of top 10 highly locally cited articles within AI for breast cancer detection and survival prediction research from 2000 to 2021.

Document	Journal	DOI	Year	Local Citations	Global Citations
Delen, Walker, and Kadam, 2005	Artif Intell Med	10.1016/j.artmed.2004.07.002	2005	65	539
Akay, 2009	Expert Syst Appl	10.1016/j.eswa.2008.01.009	2009	64	367
Zheng, Yoon, and Lam, 2014	Expert Syst Appl	10.1016/j.eswa.2013.08.044	2014	58	214
Kooi et al., 2017	Med Image Anal	10.1016/j.media.2016.07.007	2017	55	387
Arevalo et al., 2016	Comput Meth Prog Bio	10.1016/j.cmpb.2015.12.014	2016	48	172
Setiono, 2000	Artif Intell Med	10.1016/S0933-3657(99)00041-X	2000	46	140
Karabatak and Ince, 2009	Expert Syst Appl	10.1016/j.eswa.2008.02.064	2009	44	236
Araújo et al., 2017	Plos One	10.1371/journal.pone.0177544	2017	44	243
Cheng et al., 2006	Pattern Recogn	10.1016/j.patcog.2005.07.006	2006	40	303
Dheeba et al., 2014	J Biomed Inform	10.1016/j.jbi.2014.01.010	2014	39	170

### Conceptual knowledge structure analysis

#### Keyword analysis

In the current section, we apply the keyword analysis and keyword co-occurrences to analyze the research trends and developments in AI for breast cancer detection and survival predictions to display the research gaps in the literature and detect potential future research trends in AI for breast cancer detection and survival prediction field. The top fifteen keywords are highlighted in **Supplementary Figure S4**; with 805 occurrences, the keyword “breast cancer” is the most frequently occurring keyword, followed by ML (282), classification (281), DL (276), and feature selection (163). Furthermore, the correlation between AI and Breast cancer diagnosis and prognosis research can be mapped using the word growth graph shown in **Supplementary Figure S5**. As observed from the word growth graph, the occurrence per year of the main keywords, which are all the tools of AI for the earlier diagnosis of breast cancer, have grown progressively over time, namely breast cancer, DL, ML, feature selection, and classification. However, some of them, like “breast cancer, classification, ML, and DL,” grew more dynamically than other keywords. For example, in terms of cumulate occurrence in 2000, keywords breast cancer, machine learning, classification, feature selection, and deep learning were zero, one, three, one, and zero, respectively. Whereas in the year 2021, the keywords with the highest increase in occurrences from the year 2000 to 2021 were: Breast cancer (777), ML (275), classification (274), DL (258), and feature selection (162).

**Figure 3:**
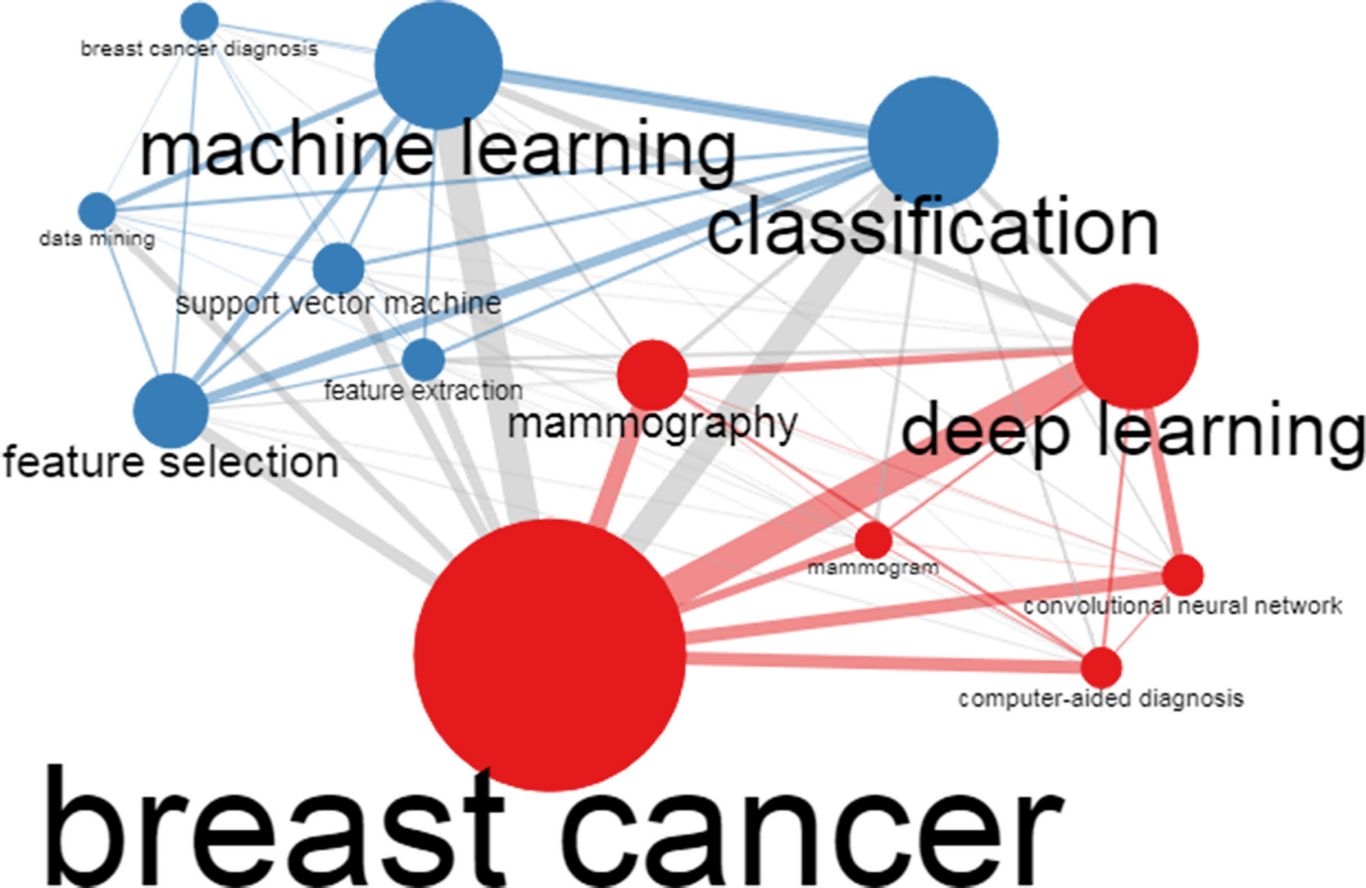
A co-occurrence network analysis of author keywords.

In addition to the author’s keyword analysis, the authors’ keywords co-occurrences were analyzed using biblioshiny. The Co-occurrence network can enable us to understand the topics covered by a research field and define the most critical and recent fronts (issues). It could also help us understand the evolution of the issues over time. The outcome of the Co-occurrence network study is presented in **Figure 3**. In **Figure 3**, the node size (keyword) represented by a dot in the network displays the number of occurrences (keywords). For instance, Breast cancer is the maximum size node, confirming that breast cancer is the most frequent keyword. In this regard, we can observe from **Figure 3** that the author’s keywords are DL, ML, and classification, the highest frequency of occurrence after breast cancer. Likewise, the width of edges linking other nodes shows the occurrence of keywords employed concurrently in the research publications present in our metadata. In this context, we observe that the author keywords “breast cancer and DL” followed by “breast cancer and ML,” “breast cancer and classification,” “breast cancer and convolutional neural network (CNN),” and “breast cancer and computer-aided diagnosis (CAD)” have the most co-occurrences in current bibliometric literature.

#### Keywords evolution trends

**Figure 4:**
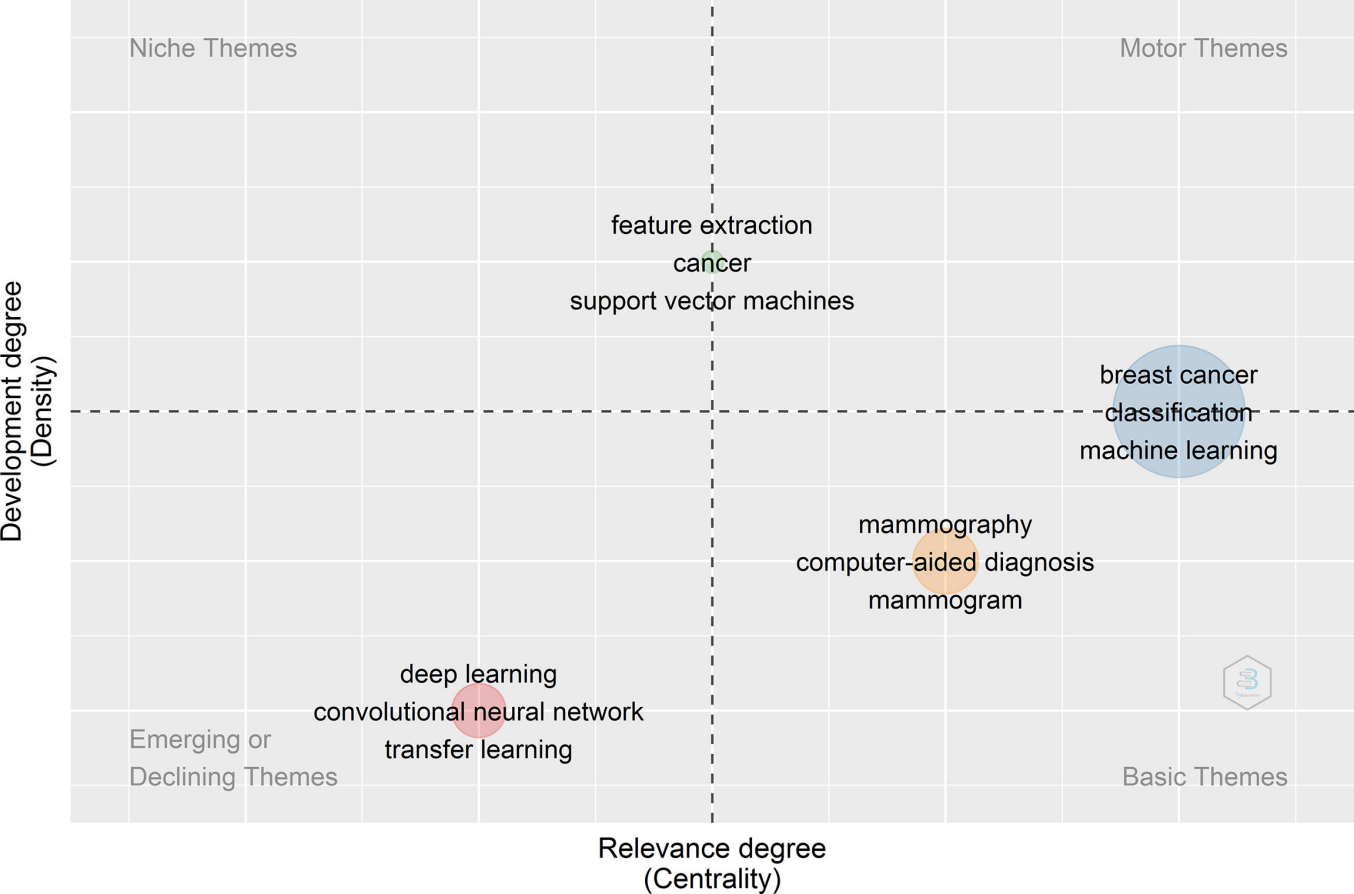
Thematic map of author's keywords.

Applying a clustering algorithm to the keywords network makes it possible to highlight different themes of a given domain. Each cluster/theme can be represented on a particular plot, known as a strategic or thematic map (27). In a thematic map, each bubble represents a network cluster. The bubble name is the word belonging to the cluster with the higher occurrence value. The bubble size is proportional to the cluster word occurrences, and the bubble position is set according to the cluster callon centrality and density. The callon centrality can be read as the importance of the theme in the entire research field, and callon density can be read as a measure of the theme’s development. Therefore, thematic maps were constructed to reveal the evolution of the keyword trends, as shown in **Figure 4**. The thematic map consists of four quadrants: The first quadrant from the right top corner signifies the thematic keywords belonging to motor themes, representing well-developed themes related to the Application of AI in breast cancer diagnosis and prognosis research.

The second quadrant represents the niche themes, which represent themes that have good internal development. The third quadrant represents the thematic keyword belonging to weakly developed, emerging, or declining themes. Finally, the fourth quadrants represent thematic keywords belonging to basic and transversal themes with weak internal development. For example, in **Figure 4**, the thematic analysis of the data obtained from 2000-2021, we observed that breast cancer classification and machine learning are both well developed and essential for the conceptual structure of the research field (AI for Breast Cancer Diagnosis and Prognosis). On the other hand, mammography, CAD, and mammogram are the themes that are important but less developed as compared to themes of the first quadrant (Motor themes). The themes such as feature extraction, cancer, and SVM have good internal development but unimportant external ties with the other themes, so they have a marginal role in the given scientific field. It is worth mentioning that the primary/transversal themes and the motor themes are considered those that support the development and strengthening of an area of knowledge (AI for breast cancer diagnosis and prognosis) due to their centrality and density.

**Figure 5:**
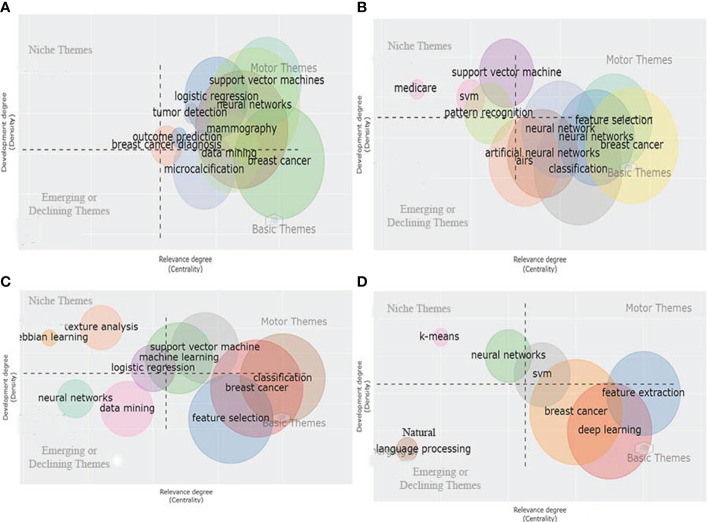
**(A–D)** Sankey diagram based on keyword thematic evolution from 2000 to 2020.

On the other hand, DL, CNN, and Transfer Learning (TL) represent the emerging or declining themes with a weak internal development degree and are marginally crucial for developing the given scientific field. Next, the thematic evolution of the keywords from 2000 to 2021 is analyzed based on the keyword thematic map and Sankey diagram shown in **Supplementary Figure S6** and **Figures 5A–D**, respectively. According to the Sankey diagram and keywords thematic map as shown in **Supplementary Figure S6** and **Figures 5A–D**, we observe that from 2000 to 2015, studies were more focused on applying ML tools to detect metastatic breast cancer masses from ultrasound breast images. However, during the last five to six years, the implementation of DL techniques to improve the accuracy of detecting suspicious cancerous breast masses using ultrasound or MRI images of breast masses has paved the way for earlier detection of breast cancer. Moreover, studies have shown that the role of Natural Language Processing (NLP) has great potential in predicting metastatic breast cancer recurrence.

#### Multicorrespondence analysis and clustering map of words

**Figure 6:**
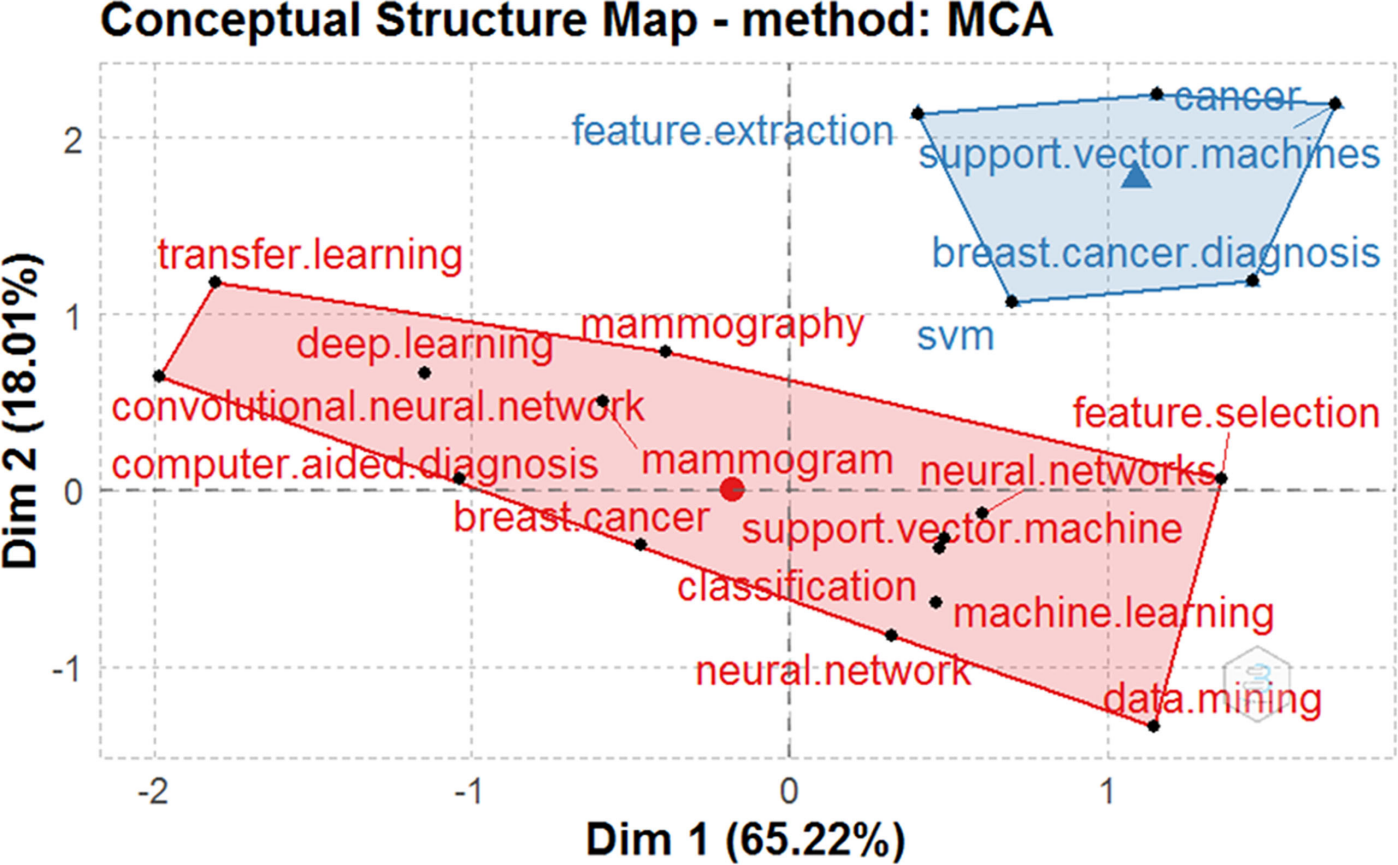
Factorial analysis of the author keywords constructed using MCA and hierarchical clustering techniques.

Similarly to the network analysis, we applied the factorial analysis (data reduction technique) to study the sub-topics related to the implementation of AI in breast cancer detection and survival prediction research, as represented in **Figure 6**. The factorial analysis was performed using the multiple correspondence analyses as the dimensionality reduction technique and hierarchical clustering as the clustering algorithm to group related terms close to each other. Through the factorial analysis, the nodes with the same color constitute a cluster that depicts their central research theme (main topic) inferred from their respective sub-topics (nodes) within a given cluster. Further, the association between two nodes is dependent on proximity between the nodes. The closer the two nodes’ proximity, the more significant the articles treat them together. Nodes with lower proximity are pulled together while nodes with high proximity are distant, thereby attaining discrete clustering among keywords. The map’s origin for each cluster in the conceptual structure map represents the average position of all column profiles and, therefore, represents the center of the research field.

The conceptual structure analysis using factorial analysis reveals that the two subfields were identified in the scientific field of AI for breast cancer detection and survival predictions. The two main subfields are as follows:

1. Red cluster grouping together author keywords: breast cancer, CAD, neural network (NN), data mining (DM), CNN, TL, DL, mammography, mammogram, SVM, classification, ML, and feature selection. The factorial analysis shows that the keyword “breast cancer” occupies a more central position in the red cluster. Thus, we can conclude that breast cancer is the red cluster’s most common and significant topic.

2. Blue cluster grouping the author keywords: SVM, breast cancer diagnosis, SVM, cancer, and feature extraction. The factorial analysis shows that the keyword “cancer” occupies a more central position in the blue cluster. Thus, we can conclude that cancer is the most common and significant keyword in the blue cluster.

#### Multicorrespondence analysis and clustering most contributing documents

The graphical map shown in **Supplementary Figure S7** allows us to identify the link between the topics and the related documents. The map plots the documents associated with the highest total contribution. The total contributions measure each document’s weight in the information summarized by the two axes. The colors represent the clusters to which each record belongs. The most contributing documents related to the blue and the red cluster are shown in **Supplementary Figure S7** and tabulated in **Table 5**. We can observe from the data available from the red cluster that the article published by Chougrad H, 2018 (34), entitled “Deep Convolutional Neural Networks (DCNN) for breast cancer screening” published in Compt Meth Prog Bio, is the most contributing paper followed closely in the second position by Masud M, 2020 (35) entitled “CNN-based models for diagnosis of breast cancer” published in Neural Computing Application.” In the same context, the article authored by Murtaza G, 2020 (36), entitled “Breast Cancer Multi-classification through Deep Neural Network (DNN) and Hierarchical Classification Approach,” published in Multimedia Tools and Applications, is the third most contributing paper. Finally, the article “MitosisNet: End-to-End Mitotic Cell Detection by Multi-Task Learning,” published in IEEE Access and authored by Alom MZ, 2020 (37), is the fourth most contributing document on the associated topics with the red cluster. The article entitled “Development of an intelligent CAD system for mass detection in mammographic images,” published in IET Image Processing, authored by Andreadis T in 2020 (38), is the most contributing paper on the topics related to the blue cluster. In addition, the articles written by Salama WM, 2020 (39) and Eltrass AS, 2020 (40) were the second and third most contributing paper in the area of research related to the blue cluster.

**Table 5:** Highly contributing Articles by clusters obtained using Multicorrespondence Analysis.

Cluster	Documents	Article tile	Journal	Contribution
**Red (I)**	Chougrad, Zouaki and Alheyane, 2018	Deep Convolutional Neural Networks for breast cancer screening	Computer Methods and Programs in Biomedicine	1.38
	Masud, Eldin Rashed, and Hossain, 2020	Convolutional neural network-based models for diagnosis of breast cancer	Neural Computing Application	1.02
	Murtaza, Shuib, Mujtaba, et al., 2020	Breast Cancer Multi-classification through Deep Neural Network and Hierarchical Classification Approach	Multimedia Tools and Applications	1.02
	Alom et al., 2020	MitosisNet: End-to-End Mitotic Cell Detection by Multi-Task Learning	IEEE Access	1.01
**Blue (II)**	Andreadis et al., 2020	Development of an intelligent CAD system for mass detection in mammographic images	IET Image Processing	4.23
	Salama, Elbagoury, and Aly, 2020	Novel breast cancer classification framework based on deep learning	IET Image Processing	4.16
	Eltrass and Salama, 2020	Fully automated scheme for computer-aided detection and breast cancer diagnosis using digitized mammograms	IET Image Processing	3.82

#### Multicorrespondence analysis and clustering most cited documents

The graphical map in **Supplementary Figure S8** allows us to identify the link between the topics and the cited documents. The graphical map plots the documents associated with the highest global citations. The colors represent the clusters to which each document belongs. The most cited papers related to the blue and the red cluster are shown in **Supplementary Figure S8** and tabulated in **Supplementary Table S5**. We can observe from the data available from the red cluster that the article published by Sirinukunwattana K, 2016 (41) entitled “Locality sensitive deep learning for detection and classification of nuclei in routine colon cancer histology images” and published in IEEE Transactions on Medical Imaging is the most cited paper (557 Citations) in deep learning a subtopic associated with the red cluster. The documents authored by Delen D, 2005 (30) and Tang J, 2009 (42), are the second with 539 and the third with 443 citations, the most globally cited papers associated with subtopics of the red cluster. In the blue cluster, the article “SVM combined with feature selection for breast cancer diagnosis,” published in Expert systems with applications, authored by Akay MF, 2009 (31), is the most cited paper with 367 citations related to topics associated with the blue cluster. In addition, the articles authored by Chen HL, 2011 (43) and Stoean R, 2013 (44) were the second and third most cited documents in research related to the blue cluster.

### Intellectual knowledge structure analysis

#### Co-citation analysis

**Figure 7:**
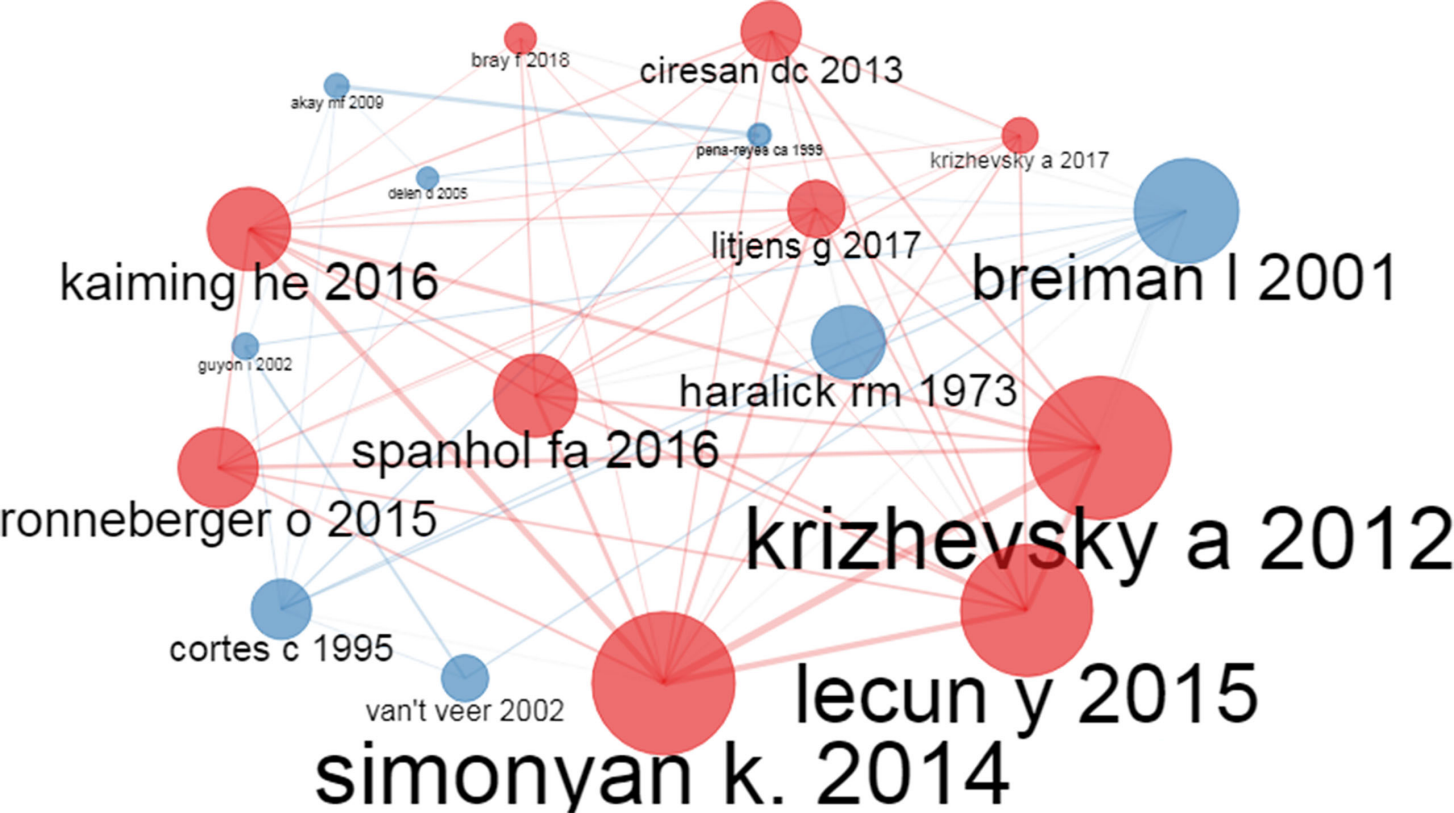
A co-citation network graph of documents.

Co-citation analysis (28) is a critical citation analysis technique in bibliometrics to show a relationship between nodes representing the author or documents (Representation of an Intellectual structure of a given research field). Here we talk about co-citation of two papers or authors when a third document or author cites both. The co-cited documents are represented as nodes, and the edges connecting the co-cited documents represent the instances of co-citation. Here the node size means the document occurrence, i.e., a paper with higher occurrence will have a correspondingly larger node size and vice versa. Moreover, the edge size is proportional to the document’s co-occurrence, i.e., records with higher co-occurrence will have a thicker edge size and vice versa. As per **Figure 7**, we can observe that the research papers by Simonyan K, 2014 (45), Kaiming HE, 2016 (46), Krizhevsky A, 2012 (47), Lecun Y, 2015 (48), Ronneberger O, 2015 (49), Spanhol FA, 2016 (50), Litjens G, 2017 (51), Bray F, 2018 (52), and Cireşan DC, 2013 (53) have been cited by other documents as well as co-cited by many source documents (documents in the dataset). Moreover, Breiman I, 2001 (54), Guyon I, 2002 (55), Haralick RM, 1973 (56), Akay MF, 2009 (31), Cortes C, 1995 (57), Delen D, 2005 (30) and Pena-Reyes CA 1999 (58) have been co-cited by other source documents. The color of the nodes in the co-citation network represents the research field to which the records belong. For example, the Red color nodes depict research in the DCNN for image classification to diagnose cancer. The blue nodes represent documents related to different ML algorithms for breast cancer diagnosis.

#### Historiography analysis

When examined over time, co-citation analysis helps detect a paradigm shift (a fundamental change in approach or underlying assumptions) and school of thought related to a particular research field (29). In **Supplementary Figure S9**, each historical path represents a research topic and its core authors and documents. Each node in **Supplementary Figure S9** represents a document (included in the analyzed collection) cited by other documents. Each edge represents a direct citation, and nodes and edges are plotted on an oriented graph where the horizontal axis represents the publication years. Here, the blue color research path represents a fundamental change in approach and school of thought related to breast cancer diagnosis and the prediction of breast cancer survivability research using AI.

From 2000 to 2015, the focus was on detecting cancer and predicting survivability using a basic ML algorithm (30–32, 43, 59–63). After that, however, the emphasis has been on using DL networks in breast cancer diagnosis and prognosis research (64). The light yellow color research path represents the automated detection and classification of masses in the mammogram. From 2000 to 2010, the focus was on using CAD for breast cancer (65, 66)). After that, however, the focus shifted to heuristic and CNN for the CAD of breast cancer. The purple-colored research path represents breast cancer diagnosis using microscopic biopsy images. From 2000 to 2015, the purple-colored research path focused on diagnosing breast cancer using the computer-aided analysis of biopsy images. After that, however, the focus shifted to CNN to diagnose breast cancer using histological images (67).

Similarly, the light red color research path represents classifying and detecting lesions in a mammogram using DL techniques. The red-colored research path originated in 2015 and continues till 2021 (68–71). Lastly, the light blue research path represents the field of breast cancer classification’s DL and TL. Although the light blue research path originated in 2016 (72), the primary contributing authors are continuously publishing in DL and TL for the diagnosis and prognosis of breast cancer (73–78).

### Social knowledge structure analysis

#### Authors’ collaboration network analysis

**Figure 8:**
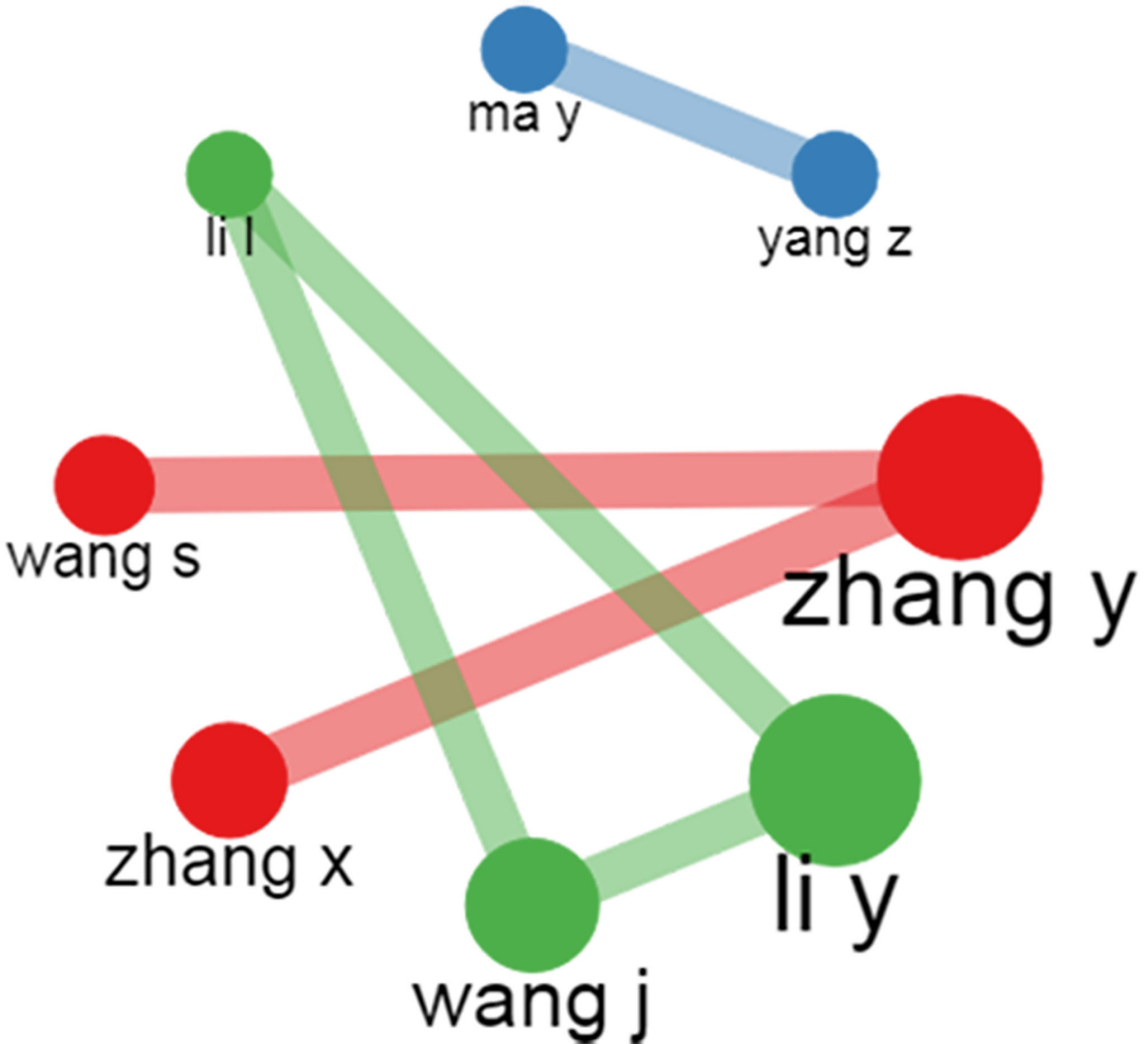
Pictorial representation of the author’s collaboration using author’s collaboration network plot.

The author’s collaboration network analysis reveals how authors interact with each other. We applied a threshold of five papers per author and represented the global collaboration of authors worldwide. **Figure 8** shows the partnership of the eight most contributing authors among the total authors in the dataset. Out of the selected fifty authors, eight authors collaborated strongly with the other authors in the dataset and had a minimum of five publications together. The thickness of the edges represents the association between the authors, and the node’s size represents the number of articles they co-authored together. For example, Wang S, Zhang Y, and Zhang X in the blue-colored research path published more papers together than other authors in the dataset. Similarly, Wang J, Li Y, and Li L in the red-colored research path published more articles than other authors in the dataset in the red-colored research field. Lastly, Ma Y and Yang Z in the green-colored research field published more articles together in the red-colored research field than the other authors in the dataset publishing article in the green-colored research field.

#### Institution collaboration network analysis

**Figure 9:**
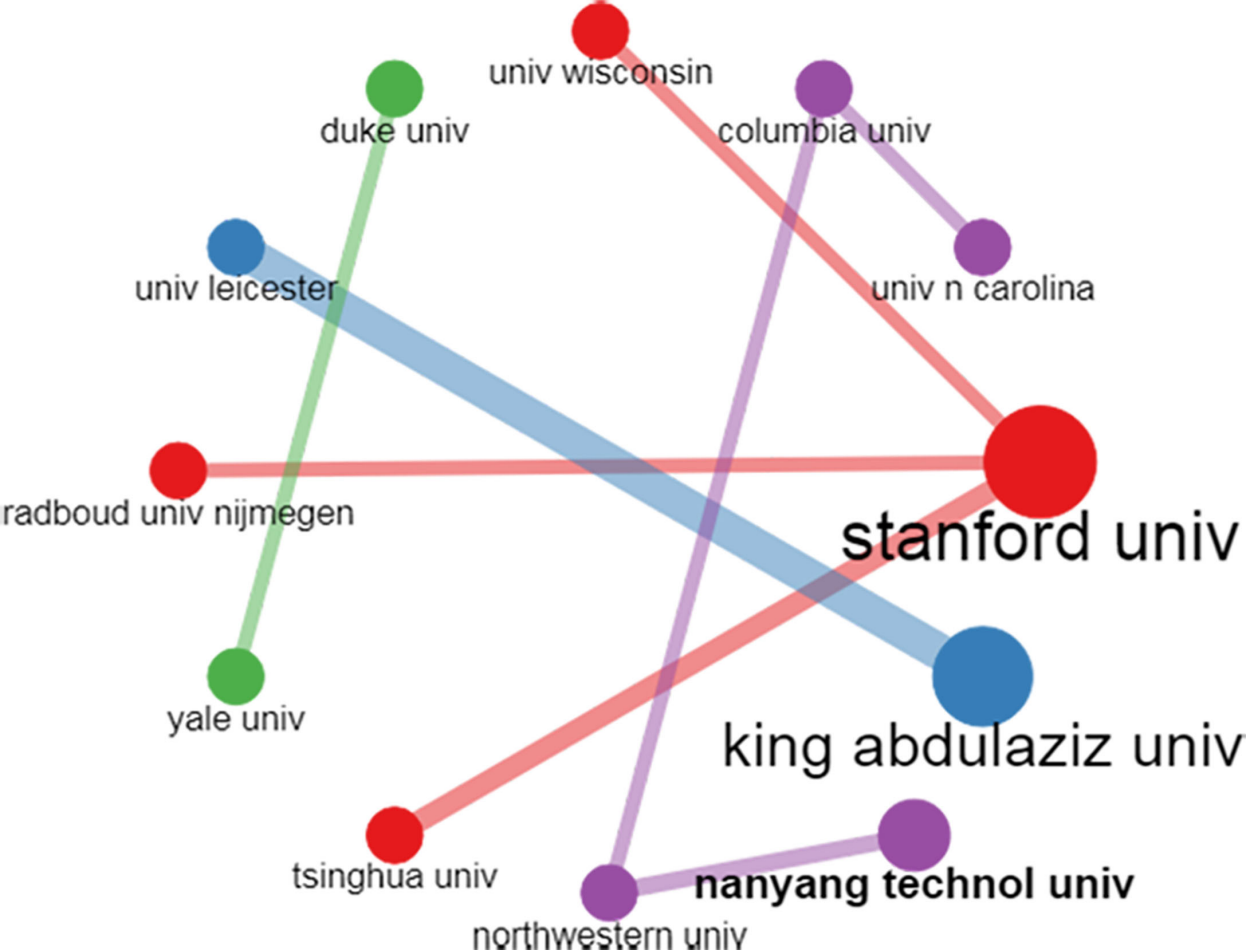
Pictorial representation of the institution’s collaboration using the institution collaboration network map.

The Institution collaboration network analysis reveals how institutions interact with each other. We applied a threshold of two or more edges and represented the global collaboration of institutions worldwide. The thickness of the edges represents the association between the institutions, and the node’s size represents the number of articles they collaborated on. Among the total institutions listed in the dataset, **Figure 9** shows the collaboration of the most collaborating institution. For example, the King Abdulaziz University of Saudi Arabia and the University of Leicester University had the maximum number of collaborated research in AI for breast cancer diagnosis and prognosis. Stanford University collaborated extensively with Radboud University and Tsinghua University in the same context.

#### Collaboration world map analysis

**Figure 10:**
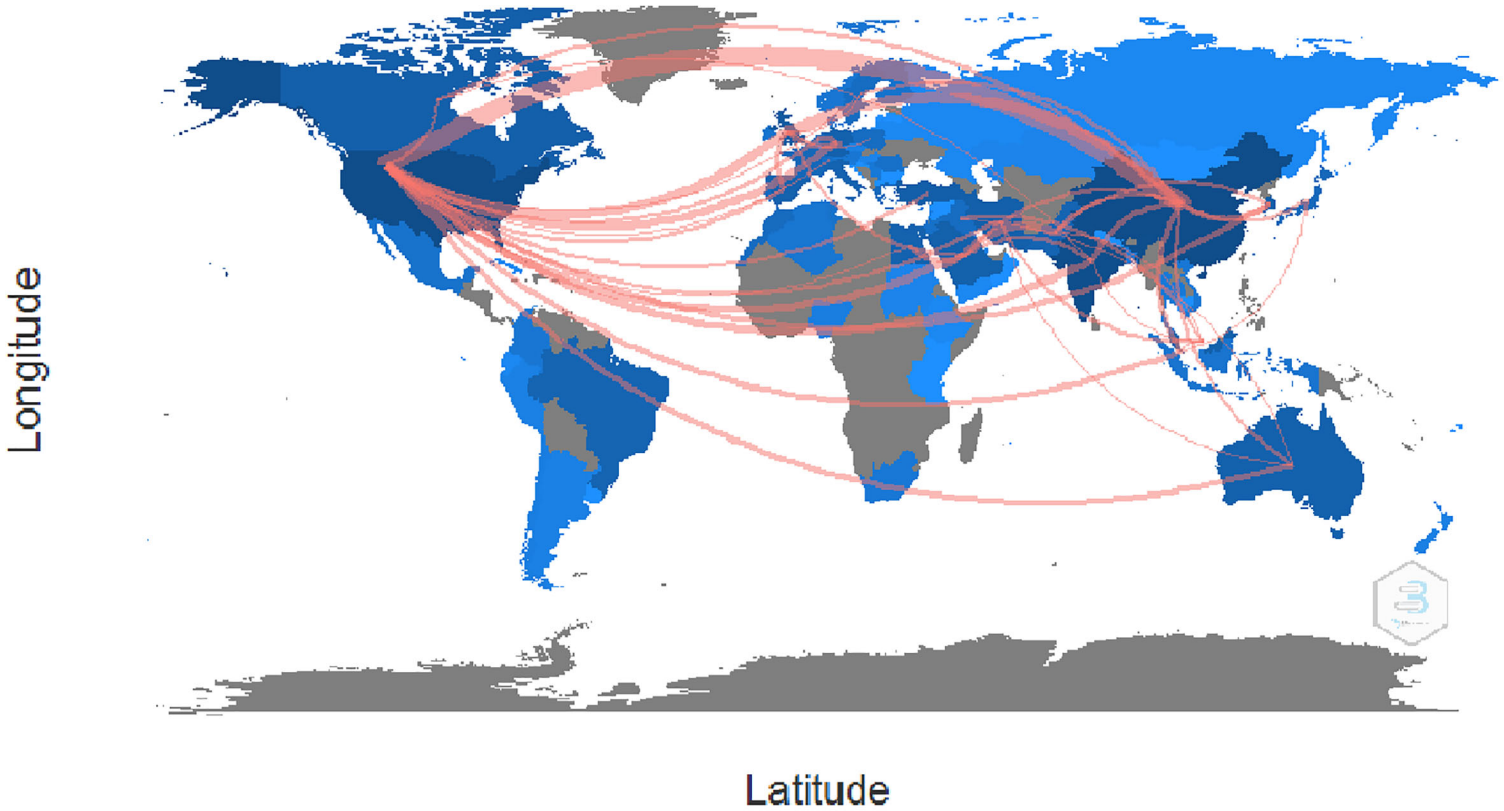
Pictorial representation of the countrywide collaboration using the country collaboration map.

As shown in **Figure 10**, the country collaboration network analysis reveals how different countries interact. We applied a threshold of five or more edges and represented countries’ collaboration worldwide. For example, from **Supplementary Table S4**, we observe that China collaborated strongly with the USA with 77 partnerships, 26 with the UK, and 10 with India in the research field of AI for breast cancer diagnosis and prognosis. In addition, the USA strongly collaborated with the UK with 20 partnerships, 13 with Germany, 13 with India, 12 with Saudi Arabia, and 11 with Korea. Concurrently, Pakistan collaborated with Saudi Arabia, the UK, and Germany in AI for breast cancer diagnosis and prognosis.

## Discussion

AI is perpetually changing the human race’s way of doing things and has been employed in many fields, including agriculture, the Internet of things (IoT), manufacturing, and intelligent healthcare. For example, since AI was introduced to detect and classify breast cancer and breast cancer patients’ survivability prediction, many academicians, scientists, and researchers have performed landmark experiments to employ different DL-based technologies for breast cancer detection and survival prediction. However, there was still a lack of a systematic evaluation of the application of DL in breast cancer diagnosis and prognosis from a bibliometric perspective. In particular, the existing literature did not conclusively answer the six questions well, including 1) What are the publishing and citation trends of the research publication in AI for breast cancer detection and survival prediction, 2) Who are the most contributing authors, journals, organizations, and countries in AI for breast cancer diagnosis and prognosis, 3) What are the publication patterns and most frequently used keywords of the articles published in AI for Breast Cancer diagnosis and prognosis, 4) What are the collaboration networks of AI research in breast cancer diagnosis and prognosis, 5) What are the thematic trends of the Application of AI in breast cancer diagnosis and prognosis research and development, and 6) What are the main open areas of challenges and the corresponding solutions for future research work in AI for breast cancer research. To address the gap in the knowledge structure in AI for Breast cancer diagnosis and prognosis, the current data and the related systematic bibliometric review methods to address the field of research are discussed. The present study depicts the research hotspots trends, publication patterns in different countries and journals, the author’s contribution and collaboration, and collaborations between countries and their institutions on AI for breast cancer diagnosis and prognosis research.

China is most productive in publishing research articles on AI for breast cancer diagnosis and prognosis research, followed closely by USA and India, respectively. While the USA has the most significant global influence based on the total citation indicators, and Netherland, in terms of average article citation, is the most influential country in research regarding the implementation of AI in breast cancer diagnosis and prognosis research. Furthermore, China is strongly collaborative with the USA, followed by the UK. Stanford University and National Taiwan University are the most relevant institutions in AI for breast cancer diagnosis and prognosis in the past two decades, from 2001 to 2021. The PLOS One is the most preferred periodical for researchers publishing articles on AI for breast cancer diagnosis and prognosis between the years 2000 to 2021. However, the journal “Expert Systems with Applications,” followed by the IEEE Transaction on Medical Imaging Journal, is the most influential AI in breast cancer detection and survival predictions research.

As per our bibliometric analysis, Zhang Y is the most contributing author and a prolific author publishing regularly in AI for breast cancer research. On the other hand, Chen H is one of the most influential authors, with 1302 citations and an H-index of 13. In 2017, Chen H and his team proposed a novel approach (Deep Contour-Aware Networks) for object instance segmentation from histopathological images (79). The proposed method won two histological object segmentation challenges: the 2015 MICCAI Nuclei Segmentation Challenge and the 2015 MICCAI Gland Segmentation Challenge, significantly surpassing all available techniques. Furthermore, Ramón Díaz-Uriarte and Sara Alvarez de Andrés, 2006 applied machine learning algorithms for gene selection and class prediction with microarray data (80) is the most globally cited article on AI for breast cancer diagnosis and prognosis research from 2000 to 2021. Delen et al., 2006 [30] compared three DM techniques for predicting breast cancer survivability, and as per the articles in our dataset collected from 2001 to 2022, their work is one of the most influential research (highly locally cited articles) in breast cancer research using AI techniques.

The keywords of a publication signify the main focus research areas, and the rate of recurrence of the keywords and their co-occurrences suggest the topics focused on that particular area of research. Accordingly, we found that “breast cancer,” “ML,” “classification,” “DL,” and “feature selection” are the most frequently occurring keywords based on keyword analysis. Analyzing the most relevant word data with that of top locally and globally cited literature offers a strong association between breast cancer and AI technologies, namely ML and DL, as these keywords are the most regularly used keywords in literature along with the most repeatedly mapped subject areas in articles present in our dataset. The current observation reveals that the prime focus of the researchers belonging to the medical imaging community is on solving medical imaging challenges in implementing AI techniques, namely DL and ML, for breast cancer research, especially concerning improving the accuracy of breast cancer screening and prognosis prediction of cancer patients. [34-40].

Morphological attributes of breast masses are crucial for classifying malignant masses based on texture and morphological characteristics of the breast images from benign tissues. Studies have shed light on using AI systems to extract features from breast ultrasound images. In a study by Hsu Sm et al., where texture attributes (namely, variance), morphological features, namely, a standard deviation of the shortest distance) and the nakagami parameters were combined to create a set of physical characteristics from the ultrasound images to build a classification model using fuzzy c-means (FCM) clustering algorithm that achieved a classification accuracy of 89.4% to discriminate between benign and malignant breast tissues (81). Zhang et al., in their study, developed a two-layer DL architecture by combining feature learning and selection techniques to extract Shear-Wave Elastography (SWE) features that performed better than the model build using the statistical features with an accuracy of 93.4% and an AUC value of 0.947, respectively (82). Furthermore, studies have shown that CAD systems, when employed to analyze the ultrasound features, enhance the diagnostic performance of inexperienced and experienced physicians (83, 84).

Moreover, the most crucial part of various diagnostic systems and human breast cancer diagnosis is the ability to classify benign breast masses from malignant breast tissues. In this context, to allow radiologists and physicians to reach a reliable conclusion in a short time regarding suspicious breast masses, AI systems have been developed gradually during the last two decades to classify benign and malignant breast masses. Several studies have used different deep learning architectures to classify malignant and benign breast lesions based on breast ultrasound images. To discuss a few DL-based studies, namely Becker AS et al., in 2018 (85) compared the performance of DL-based software for classifying malignant from benign breast tissues with three subjects with variable expertise (a trained medical student, a resident, and an experienced radiologist) in screening breast cancer using breast ultrasound images. The finding was encouraging as the DL software trained using a few hundred samples (553 benign and 84 malignant) showed comparable accuracy in classifying malignant from benign breast tissues compared to the experienced radiologist.

Moreover, the performance of the CNN-based system was better than the medical student trained using the same training data (n= 445, i.e., 70% of the total data). These findings showed that DL-based models could mimic a human decision-making process. Furthermore, in another study by Cirtisis A et al., in 2019 (86), the dCNN method achieved a classification accuracy of 95.3%, which was better than 94.1% obtained by a radiologist on the external dataset comprising ultrasound images of breast lesions. These studies have shown that AI-based tools can shorten the diagnosis time of experienced doctors (radiologists) and enhance the diagnostic capability of inexperienced doctors. Moreover, our claims of the correlation between breast cancer and AI tools can also be interpreted from the cumulative occurrence word growth graph of keywords from 2000 to 2021. We can conclude from the observation made from the word growth graph that a strong correlation between the keywords, namely “breast cancer,” “ML,” “classification,” “DL,” and “feature selection,” exists. Moreover, due to the increasing implementation of AI, particularly DL in breast masses medical image analysis for the detection of cancer, these keywords form a significant portion of the trending topics in AI for the earlier detection and survival prediction of breast cancer and breast cancer patients, respectively, during the last five years (3, 32, 33, 39, 50, 67–72, 74, 76–78, 87–90).

The conceptual structure map obtained using the factorial analysis reveals that the last two decades have shed light on AI sub-topics: CNN, TL, DL, NN, SVM, classification, ML, and feature selection. While the keywords, namely, CAD, mammography, and mammogram, represent sub-topics related to breast cancer diagnosis and detection. Consequently, we can say that the red cluster contains keywords that highlight AI techniques’ application in breast cancer diagnosis and prognosis. Moreover, with its fast computing capability, and good result reproducibility with minimum efforts, AI has shown great potential in providing fact-based and helpful information to doctors in the diagnosis of breast cancer, thereby reducing the load of medical practitioners and the amount of incorrect breast cancer analysis (91, 92). Intuitively, the high number of quality publications published related to topics in the red cluster as compared to the blue cluster can be dedicated to the increasing role of ML and DL techniques, namely, CNN (34-35, 47, 50, 68, 70, 73, and 116), NN (16, 21, 36, 53, 60–63, 93), SVM (31, 32, 43, 44, 55), feature selection (31, 43, 44), and classification (50, 59, 63, 67–69, 71, 73, 75, 79, 90) in medical image analysis task.

TL is based on applying established ML and DL approaches that implement previously learned knowledge to solve novel problems more accurately and effectively (94, 95). Hyunh et al. first applied the TL technique in 2016 (96) for breast cancer imaging, using the well-defined CNN models: ResNet, GoogLeNet, AlexNet, VGGNet, and Inception, to solve image classification tasks that were trained on natural image database, ImageNet (97). Next, Yap et al., 2018 (98) proposed implementing a deep neural learning approach for breast cancer diagnosis —with a pre-trained CN, AlexNet, using three different methods— a U-Net model, a transfer learning method, and a patch-based LeNet approach. Later, Byra et al. in 2019 (99) developed a neural TL methodology for classifying breast lesions using ultrasound images. Succeeding the previous works, many studies were published in implementing TL techniques for breast detection using an ultrasound imaging approach (100, 101). Though TL approaches have continually been improving in the context of breast ultrasound analyses for breast cancer detection, there is always room for improvement (102, 103).

The CAD system for breast cancer diagnosis and prognosis has been extensively implemented (104). Relevant studies have shown that CAD systems are helpful in refining descriptions of the breast lesion and enhancing the consistency of the attributes of the breast masses among ultrasound examiners, thereby helping in the decision-making (83, 84). Recently, the implementation of DL in the CAD system has shown great potential in optimizing resource allocation, relieving doctors’ workload, and thus significantly improving the detection and prognosis of breast cancer (33, 93, 105, 106). Besides, DL-based CAD systems are contributing significantly to the fields of contrast-enhanced mammography, ultrasound and Magnetic Resonance Imaging (MRI) (107, 108), ultrasound elastography (109), and digital breast tomosynthesis (88, 110). Thus, with the advancement of AI expertise, radiologists are confident of achieving more accurate classification and thereby achieving early detection, timely diagnosis, and apt treatment of breast cancer, thereby benefiting most breast cancer patients.

Further, the conceptual knowledge structure was evaluated using the co-occurrence network. Therefore, through the co-occurrence network of the author’s keyword, we determine that on recent fronts, “breast cancer and DL” (33, 39, 71, 72, 75, 77, 78, 87–90), “breast cancer and ML,” (3, 31, 32, 43, 44, 55), “breast cancer and classification,” (50, 59, 63, 67–69, 71, 73, 75, 79, 90), “breast cancer and CNN,” (34, 35, 47, 50, 67, 69, 73, 90), and “breast cancer and CAD” (7, 8, 10, 33, 42, 65, 90), with the highest total link strength depicts the multi-faceted implementation of AI in breast cancer detection and survival prediction research areas during the years 2020- 2021. Moreover, as per the analysis of the Sankey diagram and the thematic evolution of keywords from 2000 to 2021, we understand the following:

1. From 2000 to 2010, the motor theme focused more on keywords mammography (4, 6, 8, 10, and 42), and ML-related topics (31, 43, 44, 50, 59, 60, 63, 67–69, 71, 73, 75, 79, 90) for breast cancer diagnosis and prognosis. The researchers investigated several ML methods for automating mammogram image classification during this period. The major limitation of the conventional ML studies is the detection of breast masses which vary in size, making it challenging for the researcher to detect and classify suspicious malignant breast masses from benign breast masses (111, 112). Therefore, detecting suspicious breast masses was still an open challenge for future cancer detection and prognosis research studies.

2. During the last five to six years, the basic and the transversal themes show that keywords ML (31, 43, 44, 50, 59, 60, 63, 67–69, 71, 73, 75, 79, 90), DM (30), SVM (31, 32, 43, 44, 55), feature selection (31, 43, 44), and classification (50, 59, 63, 67–69, 71, 73, 75, 79, 90) have merged into a single cluster, namely breast cancer. Moreover, DL (33, 39, 71, 72, 75, 77, 78, 87, 88, 90) and feature extraction (32) have also evolved as the primary themes in the AI field for the diagnosis and prognosis of breast cancer in recent years (2015 to 2021). However, these fields are essential for applying AI in breast cancer diagnosis and prognosis research but are not well developed, and it is far from the goal of being fully integrated into the work of clinicians and large-scale application in the world. Still, we believe that with the progress of research in AI methodology, doctors will be in a position to achieve earlier detection of breast cancer with higher accuracy and precision.

3. NLP, another emerging area of research in recent years, has a potential role in harvesting important clinical attributes unexplored within electronic medical registers. Therefore, by developing the NLP system, researchers in the coming years can use the information present in an electronic record on cancer outcomes and treatment to find individual patient timelines of metastatic breast cancer relapse (113, 114).

As per the co-citation analysis, we can say that documents by Simonyan K, 2014 (45), Krizhevsky A, 2012 (47), and Lecun Y, 2015 (48) have a higher occurrence and co-occurrence, proving that these research articles are landmark articles in applying AI to Breast cancer diagnosis. Furthermore, the historiography analysis helps detect a paradigm shift and school of thought related to AI in breast cancer diagnosis and prognosis research. Here from the historical path analysis, we observe that during the last five to six years, the focus has been on using deep learning (64, 67–72) and transfer learning techniques (75, 77, 87, 115, 118) for an image-based detection of breast cancer and survivability prediction research.

Finally, the social knowledge structure analysis shows that authors Zhang Y& Zhang X, Zhang Y & Wang S, Wang J, Li Y, Li L, and Ma Y & Yang Z collaborated and published more papers than other authors in the dataset. Similarly, the institution collaboration network analysis reveals that the King Abdulaziz University of Saudi Arabia and the University of Leicester University had the maximum number of collaborated research in AI for breast cancer diagnosis and prognosis. In addition, Stanford University collaborated extensively with Radboud University and Tsinghua University in the same context. Finally, as per the world map collaboration analysis, we observe that the developed nations, namely China, the USA, India, the UK, and Saudi Arabia, are pivotal in promoting collaborative research on AI for breast cancer diagnosis and prognosis research through their constant search for collaboration with other countries. However, we observed that institutions in developed countries seldom take the initiative to collaborate with institutions in developing and underdeveloped economies. Instead, the developed nations tend to select equally good or better institutions than themselves as collaborators.

However, these DL and TL techniques have not been declared primary clinical protocols for clinicians to detect breast cancer and cancer patients’ survivability. Thus, the scientific community must collaborate globally to undertake the necessary medical device regulation to use deep learning technology in health care. Therefore, the current systematic bibliometric review could be a valuable resource for beginners who wish to apply DL and TL techniques for breast cancer classification, detection, and survivability through different medical imaging modalities.

### Open challenges in AI for breast cancer diagnosis and prognosis

As per the evolution of the field of AI and its application in breast cancer diagnosis and prognosis has evolved, we observe from the thematic map that during the last five to six years, the basic and the transversal themes show that keyword, DL, and TL have evolved as the primary themes in the AI field for the diagnosis and prognosis of breast cancer in recent years (2015 to 2021). However, although DL and TL themes are essential for applying AI in breast cancer diagnosis and prognosis research (70, 71, 74, 76, 88, 89), these fields have not developed enough to be used as clinically proven technology to be used by clinicians for earlier detection of cancer and cancer patient survivability predictions using histopathological images and mammograms (90, 116). Therefore, efforts have to be made by the scientific community globally to collaborate efficiently to implement DL technologies to improve the performance of breast cancer classification and detection performance. Hence, these DL techniques can be used as a primary diagnostic tool for the detection of breast cancer and survivability prediction of breast cancer patients with greater accuracy and precision.

Moreover, we observed that the developed nations’ institutions seldom take the initiative to cooperate with institutions in developing and underdeveloped countries. Instead, the developed nations tend to select equal or better institutions with infrastructure and intellects than themselves as collaborators. Therefore, a country with better infrastructure and economy should collaborate with prolific intellectuals and their affiliated institutions from developing and underdeveloped countries with funded projects to try and utilize the current technology to establish a worldwide AI-based breast cancer healthcare ecosystem. The AI-based breast cancer healthcare ecosystem will allow institutions from underdeveloped countries to significantly implement advanced DL techniques in breast cancer diagnosis and prognosis.

Clinical and image data should be shared. However, data that is demonstrative of typical breast cancer patients, annotated, structured, and ready to be used is inadequate and available in only a few institutions. Therefore new imaging repositories, such as the Health Data Research Innovation Gateway, must be set up to address this data gap. In addition, setting up new image repositories is vital for developing a data ecosystem to meet the demand for developing a novel algorithm for the earlier detection and treatment response prediction of breast cancer.

Further, it is essential to bring scientific fields together, which means a new multidisciplinary team, including clinical scientists, informaticians, and clinicians needs to be trained and developed to incorporate AI analysis into breast cancer care decisions (117).

### Limitations

Our bibliometric review has some limitations. First, we included publications available only in the English language. Secondly, we did not include electronic preprints studies published in an online open-access repository, the ArXiv. We might have skipped several publications related to AI and Breast cancer diagnosis and prognosis research; nevertheless, these electronic preprints in the online repositories are not peer-reviewed articles. Third, we only extracted and analyzed data from WOS and Scopus data from January 2000 to October 2021. So we might have missed many articles linked to AI and Breast cancer diagnosis and prognosis research published between the years November 2021 to January 2022.

## Conclusion

DL, feature extraction, and TL for breast cancer diagnosis have become basic and transversal themes in the last five to six years. However, these fields are not well developed enough to be used by clinicians for regular cancer detection and prognosis prediction. Therefore, there is urgent to convert these basic themes to motor themes and append these techniques to clinical practices as a breast cancer diagnostic or prognostic tool. Therefore, the current systematic bibliometric review could be a valuable resource for beginners applying AI to researchers on DL-based breast cancer classification through different medical imaging modalities.

## Data availability statement

Publicly available datasets were analyzed in this study. This data can be found here: Web of Science and Scopus.

## Author contributions

All authors made substantial intellectual involvement in the present study to meet the requirements as authors. First, AS and TK apprehended the study’s design. Second, AS and TK collected the data, and AS performed the research and analyzed the data. Third, TK drafted the materials and methodology and edited the figures. Fourth, AS drafted the abstract, introduction, result, and discussion. Finally, AS and TK edited the manuscript. All authors agree to be accountable for the content of the work

## Funding

This project was funded by the Deanship of Scientific Research (DSR), King Abdulaziz University, Jeddah, under grant no. (D:830-1021-1443). The authors, therefore, gratefully acknowledge DSR's technical and financial support.

## Conflict of interest

The authors declare that the research was conducted in the absence of any commercial or financial relationships that could be construed as a potential conflict of interest.

## Publisher’s note

All claims expressed in this article are solely those of the authors and do not necessarily represent those of their affiliated organizations, or those of the publisher, the editors and the reviewers. Any product that may be evaluated in this article, or claim that may be made by its manufacturer, is not guaranteed or endorsed by the publisher.

## Supplementary material

Supplementary Figure S1 Study Selection workflow.

Supplementary Figure S2 Most relevant affiliation in AI research for breast cancer detection and prognosis prediction.

Supplementary Figure S3 Source clustering through Bradford’s Law.

Supplementary Figure S4 Top fifteen keywords in AI for breast cancer detection and survival prediction research from 2000 to 2021.

Supplementary Figure S5 Keywords growth curve from 2000 to 2022.

Supplementary Figure S6 Sankey diagram based on keyword thematic evolution from 2000 to 2020.

Supplementary Figure S7 Factorial map of the documents in the red and blue clusters with the highest contributions.

Supplementary Figure S8 Factorial map of the documents in the red and blue clusters with the highest citations.

Supplementary Figure S9 Historical direct citation network analysis from 2000 to 2021.
