# Corrigendum: Evolution of research trends in artificial intelligence for breast cancer diagnosis and prognosis over the past two decades: A bibliometric analysis

**DOI:** 10.3389/fonc.2022.1061324

**Published:** 2023-01-05

**Authors:** Asif Hassan Syed, Tabrej Khan

**Affiliations:** ^1^ Department of Computer Science, Faculty of Computing and Information Technology Rabigh (FCITR), King Abdulaziz University, Jeddah, Saudi Arabia; ^2^ Department of Information Systems, Faculty of Computing and Information Technology Rabigh (FCITR), King Abdulaziz University, Jeddah, Saudi Arabia

**Keywords:** artificial intelligence, breast cancer, diagnosis and prognosis, Bibliometrix analysis, knowledge structures

In the published article, there was an error in the Funding statement, which was mistakenly included as an Acknowledgements. The corrected statement appears below:

## Funding

This project was funded by the Deanship of Scientific Research (DSR), King Abdulaziz University, Jeddah, under grant no. (D:830-1021-1443). The authors, therefore, gratefully acknowledge DSR’s technical and financial support.

The authors apologize for this error and state that this does not change the scientific conclusions of the article in any way.

