# Supplementary material for: Evolution of research trends in artificial intelligence for breast cancer diagnosis and prognosis over the past two decades: A bibliometric analysis
Source: Front Oncol. 2022 Sep 23;12:854927. doi: 10.3389/fonc.2022.854927 (PMC9578338; doi:10.3389/fonc.2022.854927)
Supplement: Supplementary file 1 [file Table_1.docx]

**Supplementary Table S1:** List of keywords

|  |
| --- |
| ( TITLE-ABS-KEY ( “Artificial intel*,”  OR  “machine learning”  OR  “deep learning”  OR  “neural network*,”  OR  “CNN,”  OR  “convolutional neural network”  OR  “SVM”  OR  “Random forest”  OR  “Logistic regression”  OR  “RNN”  OR  “LSTM”  OR  “Autoencoder”  OR  “KNN”  OR  “ANN”  OR  “DT”  OR  “NB”  OR  “KMean”  OR  “CMEAN”  OR  “hierarchical Algorithm”  OR  “Bagging”  OR  “Boosting”  OR  “Stacking”  OR  “GP”  OR  “BN”  OR  “JRip”  OR  “JELM”  OR  “ELM” )  AND  TITLE-ABS-KEY ( ( “Breast Cancer”  AND  “detection” )  OR  ( “Breast Cancer”  AND  “classif*” )  OR  ( “Breast Cancer”  AND  “prognosis detection” )  OR  ( “Breast Cancer AND “  mortality  AND risk  ") OR (“  breast  AND cancer  AND  “survival” )  OR  ( “Breast Cancer”  AND  “prediction” )  OR  ( “Breast Cancer”  AND  “Microarray Gene Expression Data” ) ) ) |
