# Supplementary material for: Evolution of research trends in artificial intelligence for breast cancer diagnosis and prognosis over the past two decades: A bibliometric analysis
Source: Front Oncol. 2022 Sep 23;12:854927. doi: 10.3389/fonc.2022.854927 (PMC9578338; doi:10.3389/fonc.2022.854927)
Supplement: Supplementary file 2 [file Table_2.docx]

**Supplementary Table S2:** Mapping of Research questions with knowledge structure, bibliometric and statistical techniques

| S# | Research Questions | Knowledge structure covered | Bibliometric techniques |
| --- | --- | --- | --- |
| 1 | What are the publishing and citation trends of the research publication in AI for breast cancer detection and survival prediction? | Intellectual structure | Annual Scientific Production, Average citation per year |
| 2 | Who are the most contributing authors, journals, organizations, and countries in AI for breast cancer diagnosis and prognosis? | Intellectual structure | Three field plot, Most relevant Authors, Co-citation, and Coupling |
| 3 | What are the publication patterns and most frequently used keywords of the articles published in AI for Breast Cancer diagnosis and prognosis? | Conceptual structure | Network Analysis, Factorial analysis, Thematic mapping, and Thematic evolution |
| 4 | What are the collaboration networks of AI research in breast cancer diagnosis and prognosis? | Social structure | Authors collaboration network, Institution collaboration network, and Country Collaboration WorldMAP |
| 5 | What are the thematic trends of the Application of AI in breast cancer diagnosis and prognosis research and development? | Conceptual structure | Thematic mapping, Thematic evolution, and Factorial analysis |
| 6 | What are the main open areas of challenges and the corresponding solutions for future research work in AI for breast cancer research? | Conceptual structure | Thematic mapping, Thematic evolution, and Factorial analysis |
