# Supplementary material for: Evolution of research trends in artificial intelligence for breast cancer diagnosis and prognosis over the past two decades: A bibliometric analysis
Source: Front Oncol. 2022 Sep 23;12:854927. doi: 10.3389/fonc.2022.854927 (PMC9578338; doi:10.3389/fonc.2022.854927)
Supplement: Supplementary file 3 [file Table_3.docx]

**Supplementary Table S3:** Data collecting Stages

| Stages | Inclusion and Exclusion criteria |
| --- | --- |
| Stage 1 |  |
| Database query  Web of Science:  Results: 1841 | **Inclusion Criteria:**   1. Keywords: (Tabulated in Table 1) 2. Document Type: Articles, Review articles, and early access articles 3. Subject Area: Computer Science 4. Source type: Journal 5. Year: 2000 to 2021 6. Language: English   **Exclusion Criteria:**   1. Document Type: Proceeding papers, book chapters, editorial materials, letters, data paper 2. Subject Area: Subject area other than Computer Science 3. Year: > 2000 4. Language: Other than English |
| Database query  Scopus  Results:  1737 | **Inclusion Criteria:**   1. Keywords: Tabulated in Table 1 2. Document Type: Articles, Review articles 3. Subject Area: Computer Science 4. Source Type: Journal 5. Year: 2000 to 2021 6. Language: English   **Exclusion Criteria:**   1. Document Type: Conference paper and review, book and book chapters, editorial materials, letters, short survey, notes, data paper 2. Subject Area: Subject area other than Computer Science 3. Year: > 2000 4. Language: Other than English 5. Source Type: Book and Book series, Conference proceedings, Trade journal, and undefined |
| Stage 2: |  |
| Combining results from both databases  Results: 2641 | Deleting duplicate studies ad obtaining the original dataset |
