# Supplementary material for: Evolution of research trends in artificial intelligence for breast cancer diagnosis and prognosis over the past two decades: A bibliometric analysis
Source: Front Oncol. 2022 Sep 23;12:854927. doi: 10.3389/fonc.2022.854927 (PMC9578338; doi:10.3389/fonc.2022.854927)
Supplement: Supplementary file 4 [file Table_4.docx]

**Supplementary Table S4** Highly collaborating countries in AI for breast cancer diagnosis and prognosis research represented by Collaboration WorldMAP

| **From**  **Country** | **To**  **Country** | **Frequency** |
| --- | --- | --- |
| CHINA | USA | 77 |
| CHINA | UNITED KINGDOM | 26 |
| USA | UNITED KINGDOM | 20 |
| PAKISTAN | SAUDI ARABIA | 14 |
| USA | GERMANY | 13 |
| USA | INDIA | 13 |
| USA | SAUDI ARABIA | 12 |
| USA | KOREA | 11 |
| CHINA | INDIA | 10 |
| UNITED KINGDOM | GERMANY | 10 |
