# Supplementary material for: Evolution of research trends in artificial intelligence for breast cancer diagnosis and prognosis over the past two decades: A bibliometric analysis
Source: Front Oncol. 2022 Sep 23;12:854927. doi: 10.3389/fonc.2022.854927 (PMC9578338; doi:10.3389/fonc.2022.854927)
Supplement: Supplementary file 5 [file Table_5.docx]

**Supplementary Table S5:** Highly cited article Articles by clusters obtained using Multicorrespondence Analysis

| Cluster | Documents | Article tile | Journal | Global Citations |
| --- | --- | --- | --- | --- |
| Red (I) | Sirinukunwattana et al., 2016 | Locality sensitive deep learning for detection and classification of nuclei in routine colon cancer histology images | IEEE Transactions on Medical Imaging | 557 |
|  | Delen, Walker, and Kadam, 2005 | Predicting breast cancer survivability: a comparison of three data mining methods | Artificial Intelligence in Medicine | 539 |
|  | Tang et al., 2009 | Computer-Aided Detection and Diagnosis of Breast Cancer With Mammography: Recent Advances | IEEE Transactions on Information Technology in Biomedicine | 443 |
| Blue (II) | Akay, 2020 | Support vector machines combined with feature selection for breast cancer diagnosis | Expert systems with applications | 367 |
|  | Chen et al., 2011 | A support vector machine classifier with rough set-based feature selection for breast cancer diagnosis | Expert systems with applications | 184 |
|  | Stoean and Stoean, 2013 | [Modeling medical decision making by support vector machines, explaining by rules of evolutionary algorithms with feature selection](https://www.sciencedirect.com/science/article/pii/S0957417412012171?casa_token=caQqcFJoo7oAAAAA:O-Ht8C3uMzUbnzJGHTapxXX_fK5ywifBRh3Al0Vznwz1SF8moy9vWXHXvBJGAVDuumcf3mlV3AXi) | Expert systems with applications | 53 |
