# Supplementary figures and images for: Evolution of research trends in artificial intelligence for breast cancer diagnosis and prognosis over the past two decades: A bibliometric analysis
Source: Front Oncol. 2022 Sep 23;12:854927. doi: 10.3389/fonc.2022.854927 (PMC9578338; doi:10.3389/fonc.2022.854927)

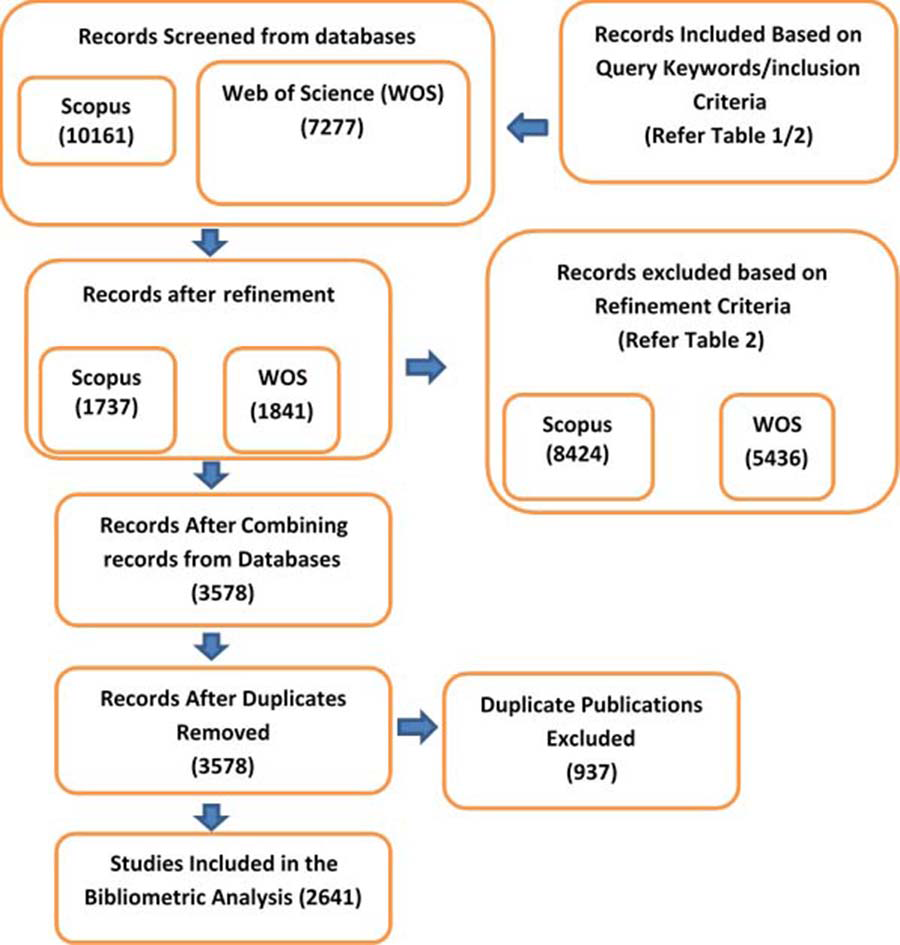

Supplement: Supplementary Figure S1 — Study Selection workflow. [file Image_1.tif]

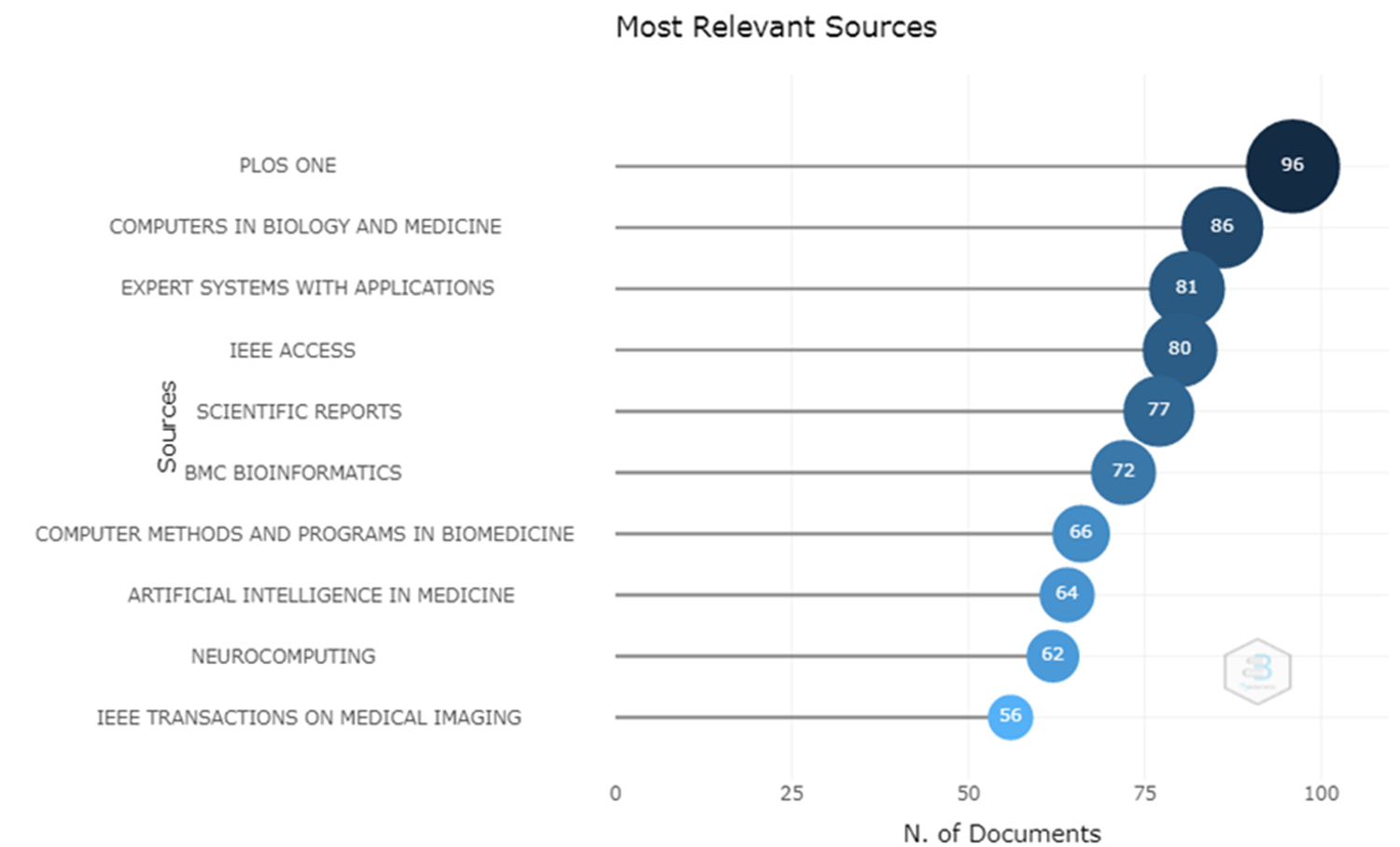

Supplement: Supplementary Figure S2 — Most relevant affiliation in AI research for breast cancer detection and prognosis prediction. [file Image_2.tif]

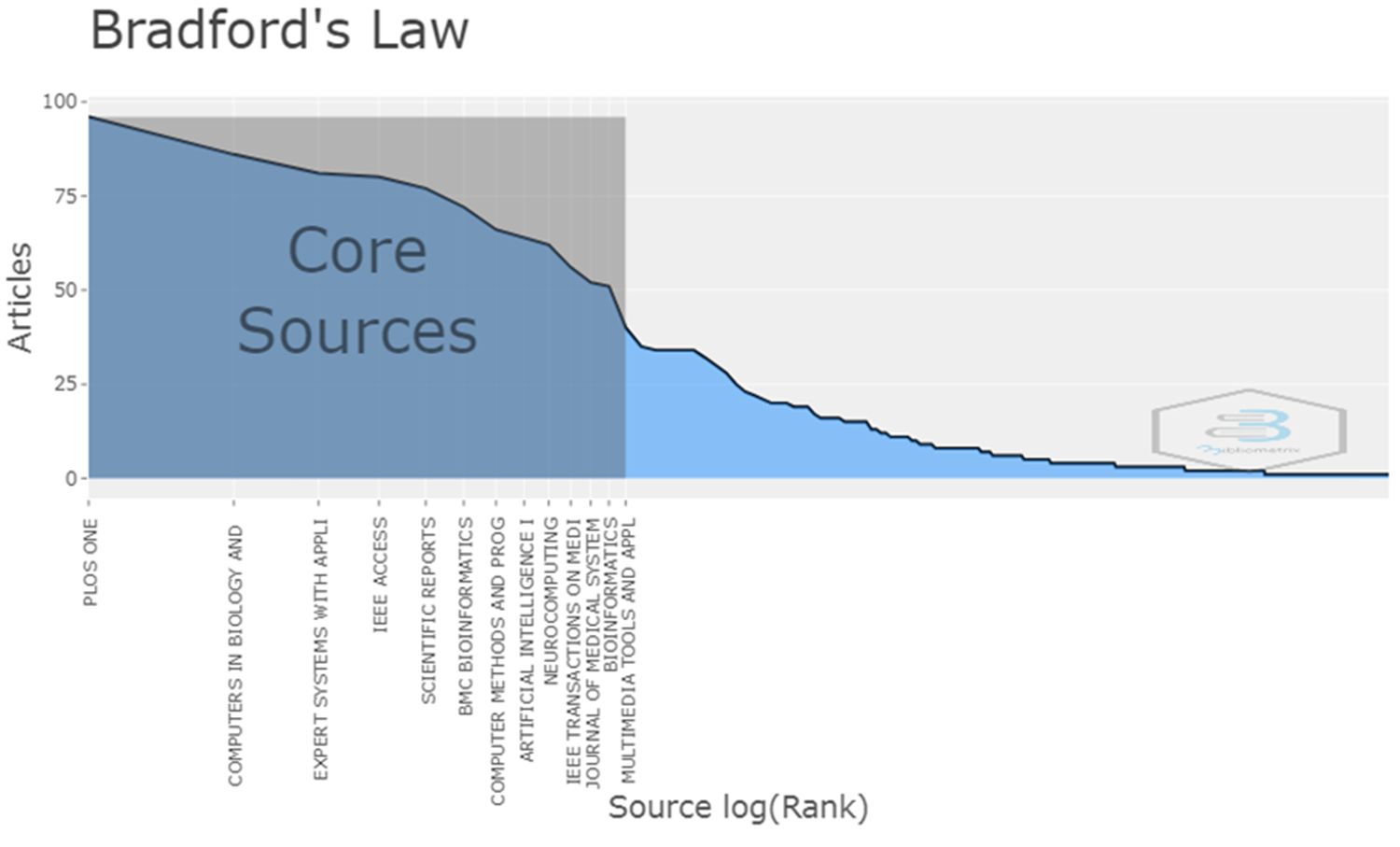

Supplement: Supplementary Figure S3 — Source clustering through Bradford’s Law. [file Image_3.tif]

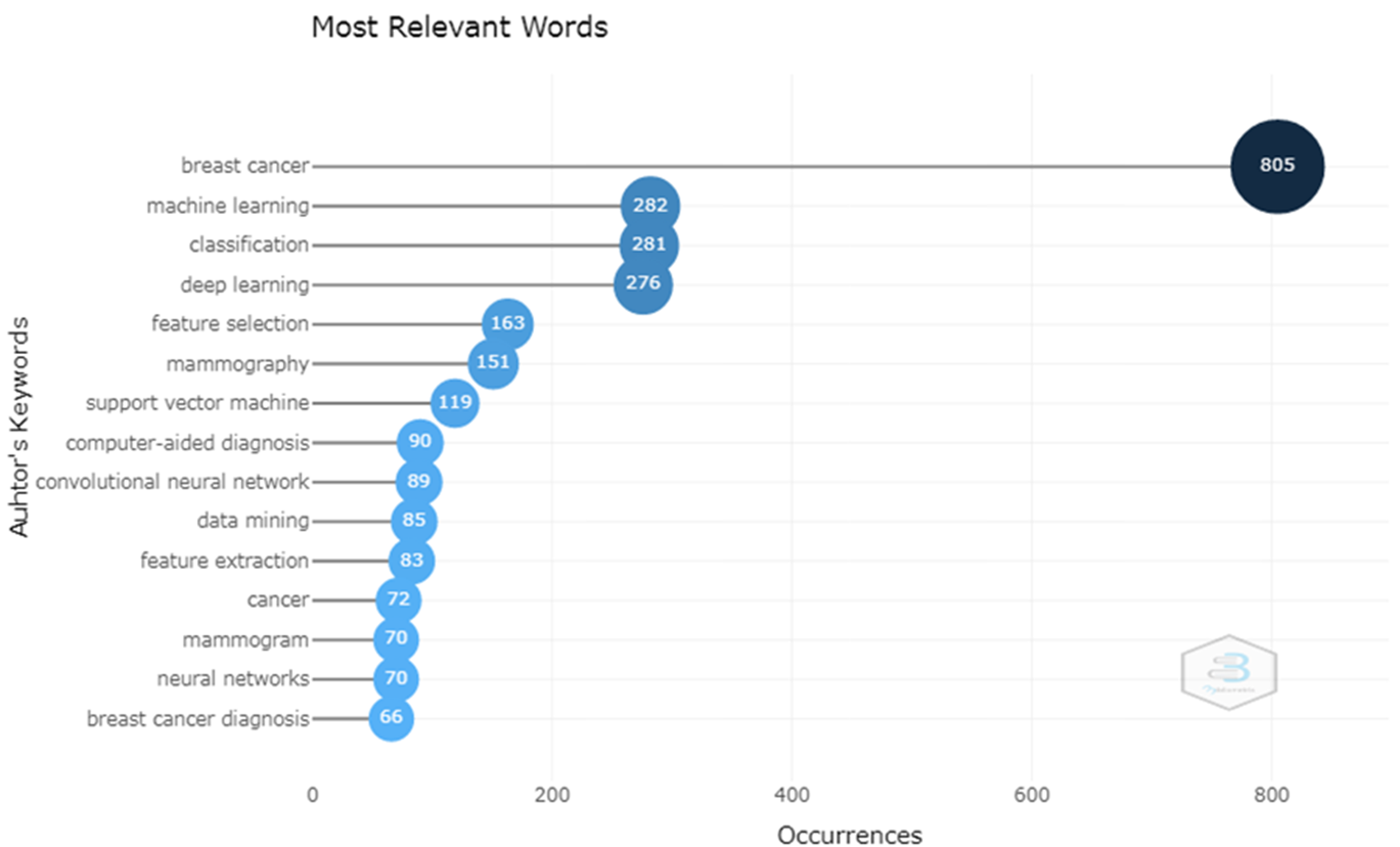

Supplement: Supplementary Figure S4 — Top fifteen keywords in AI for breast cancer detection and survival prediction research from 2000 to 2021. [file Image_4.tif]

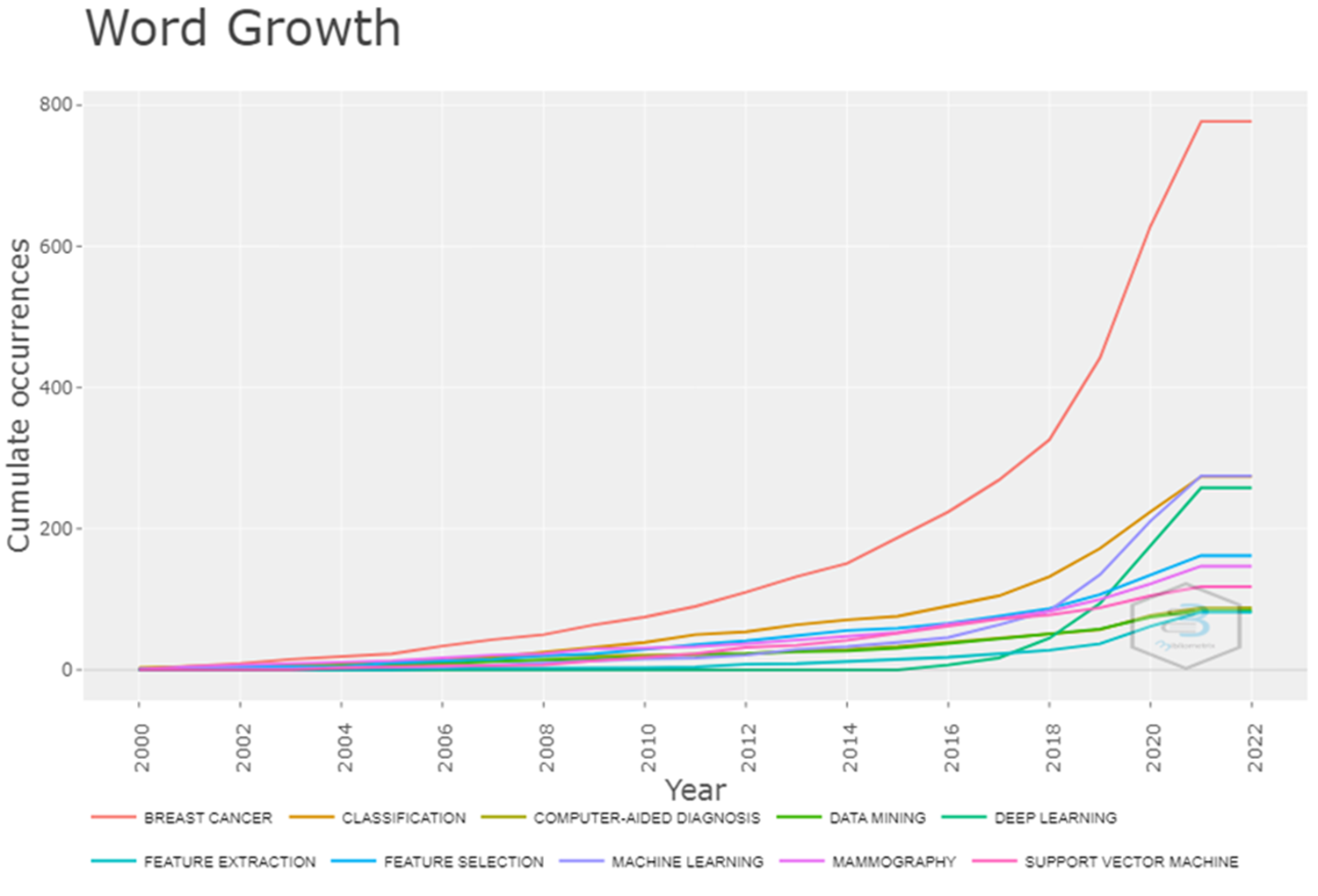

Supplement: Supplementary Figure S5 — Keywords growth curve from 2000 to 2022. [file Image_5.tif]

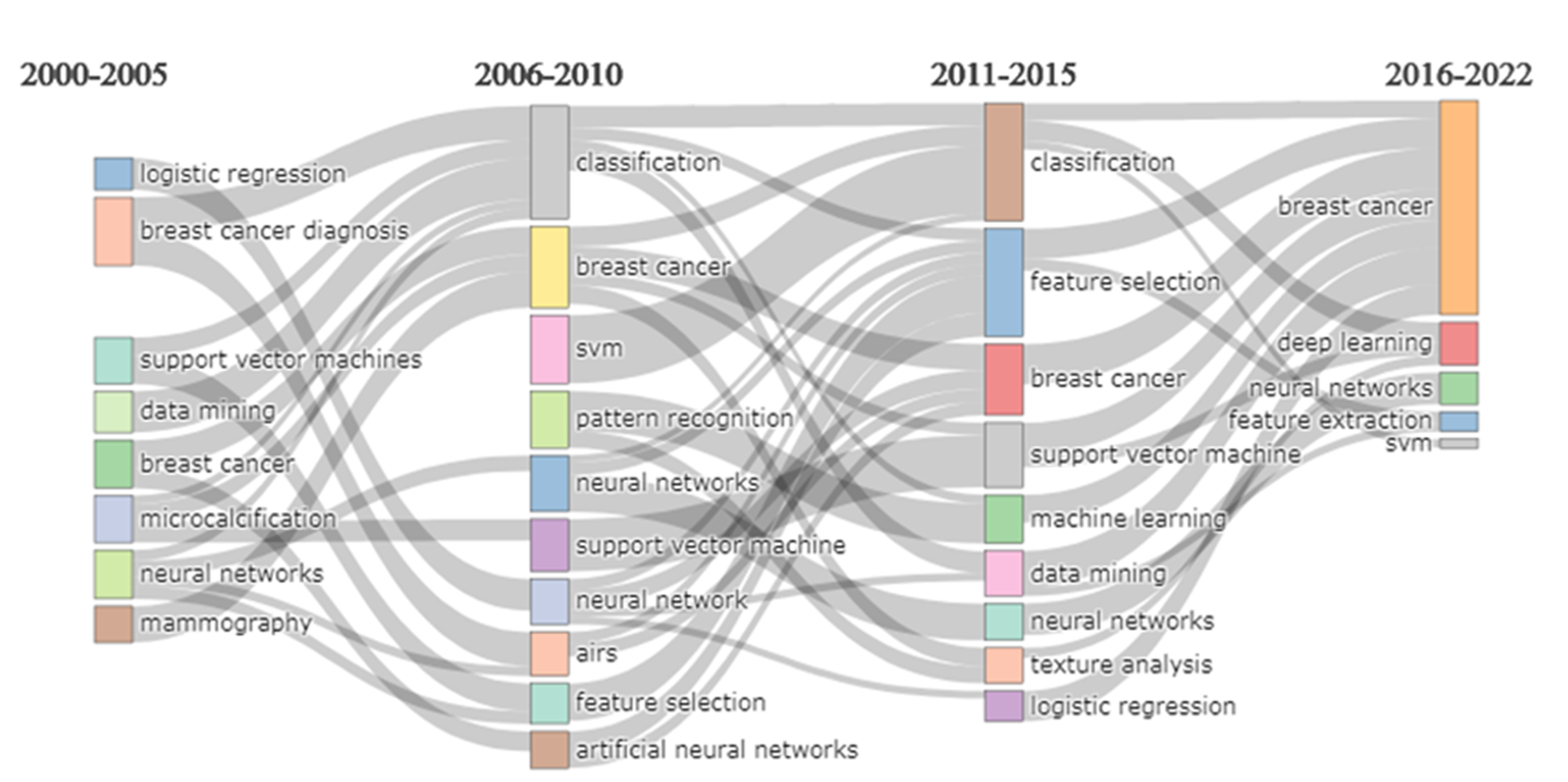

Supplement: Supplementary Figure S6 — Sankey diagram based on keyword thematic evolution from 2000 to 2020. [file Image_6.tif]

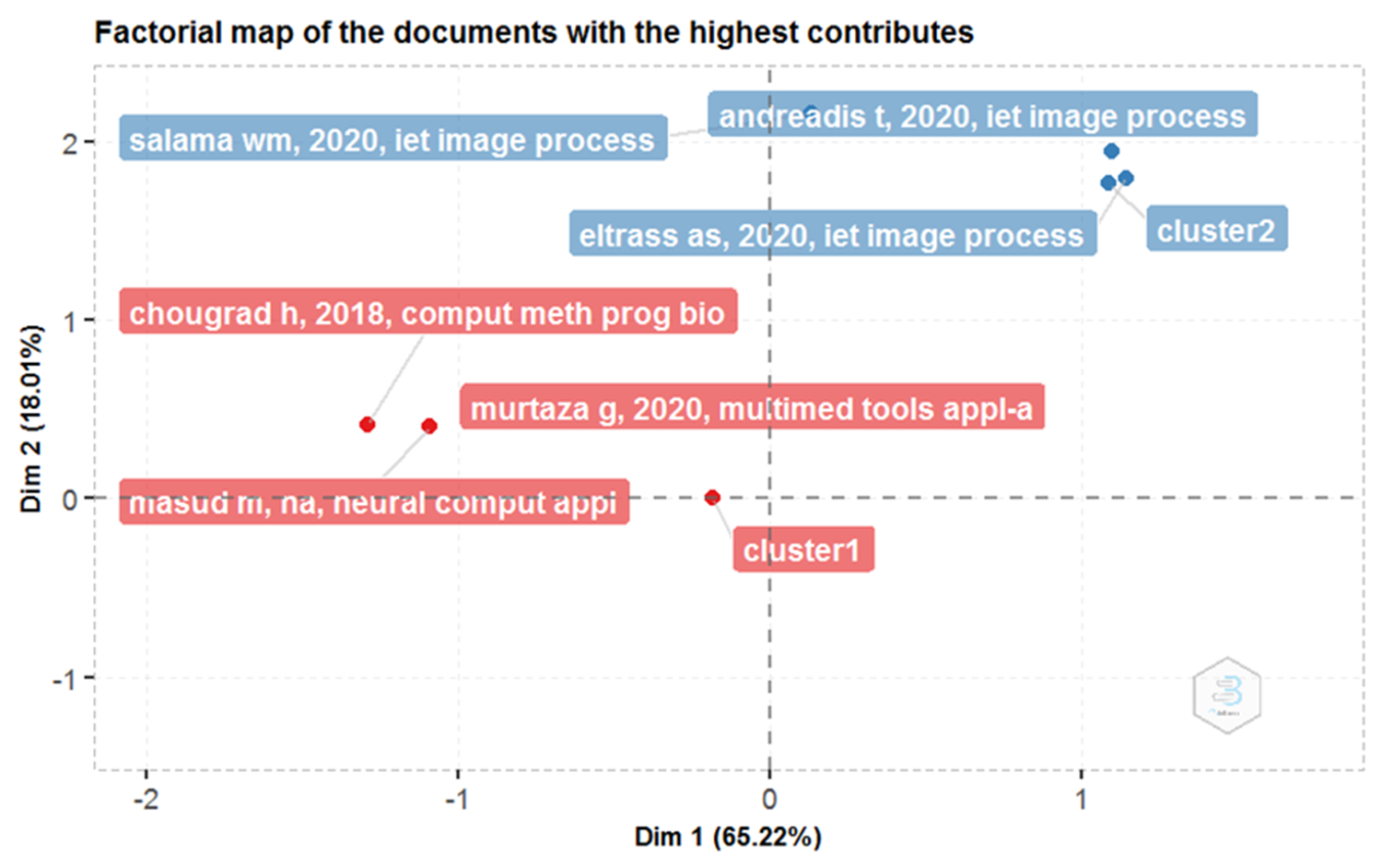

Supplement: Supplementary Figure S7 — Factorial map of the documents in the red and blue clusters with the highest contributions. [file Image_7.tif]

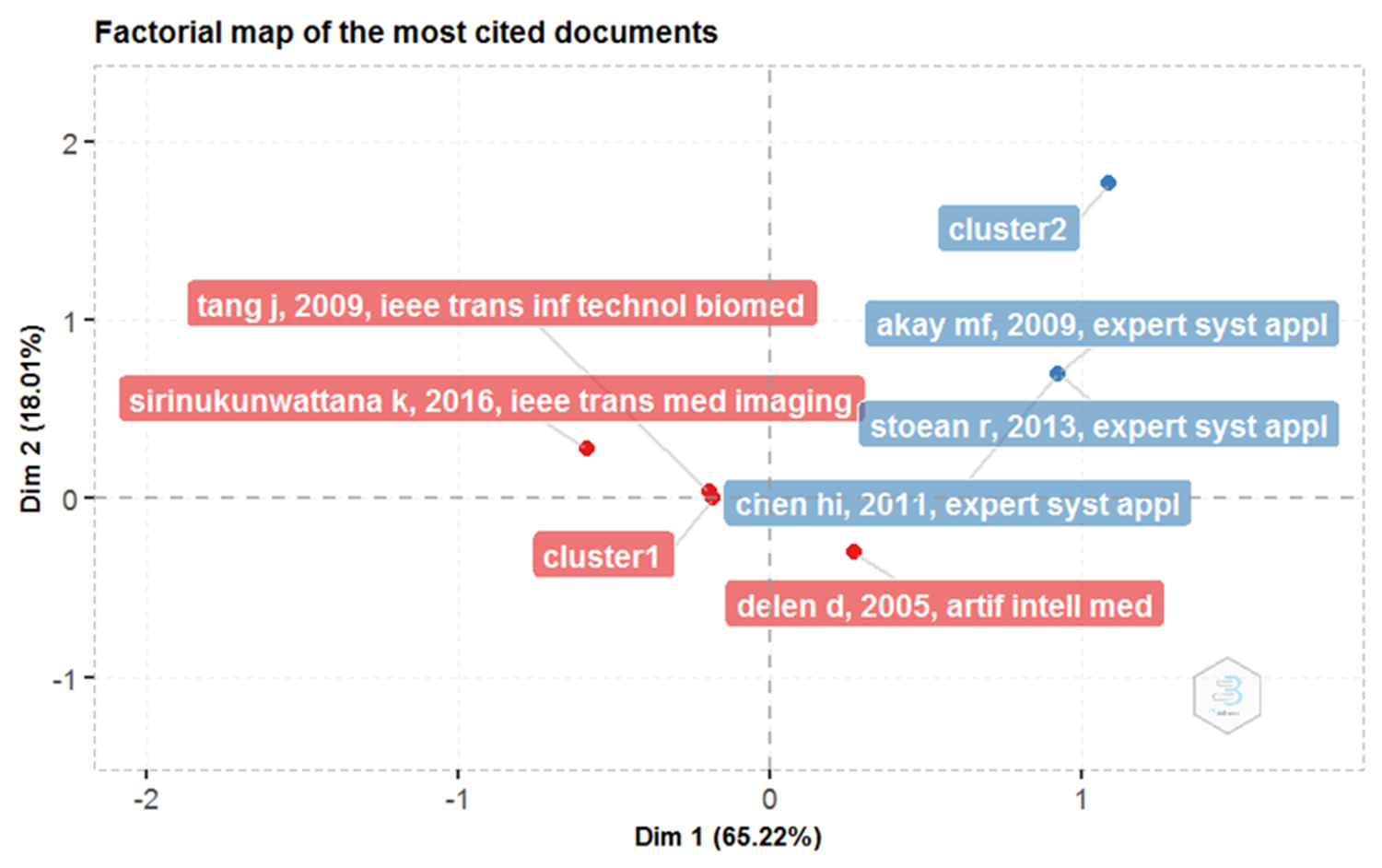

Supplement: Supplementary Figure S8 — Factorial map of the documents in the red and blue clusters with the highest citations. [file Image_8.tif]

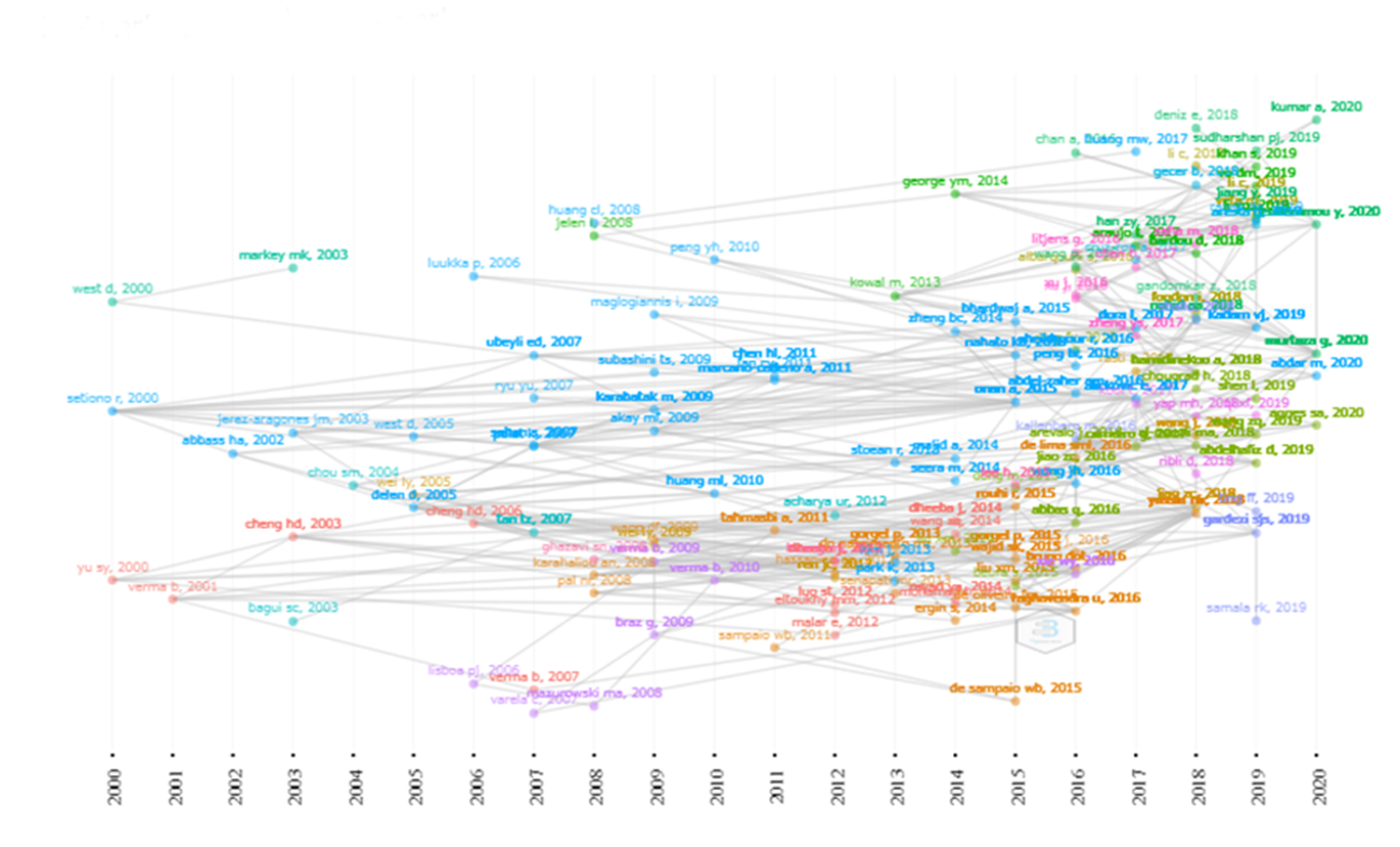

Supplement: Supplementary Figure S9 — Historical direct citation network analysis from 2000 to 2021. [file Image_9.tif]
